# Extended fiducial inference for individual treatment effects via deep neural networks

**DOI:** 10.1007/s11222-025-10624-8

**Published:** 2025-05-17

**Authors:** Sehwan Kim, Faming Liang

**Affiliations:** 1 Department of Statistics, Ewha Womans University, Seoul, 03760 Republic of Korea; 2 Department of Statistics, Purdue University, West Lafayette, IN 47907 USA

**Keywords:** Causal Inference, Deep Learning, Fiducial Inference, Stochastic Gradient MCMC, Uncertainty Quantification

## Abstract

Individual treatment effect estimation has gained significant attention in recent data science literature. This work introduces the Double Neural Network (Double-NN) method to address this problem within the framework of extended fiducial inference (EFI). In the proposed method, deep neural networks are used to model the treatment and control effect functions, while an additional neural network is employed to estimate their parameters. The universal approximation capability of deep neural networks ensures the broad applicability of this method. Numerical results highlight the superior performance of the proposed Double-NN method compared to the conformal quantile regression (CQR) method in individual treatment effect estimation. From the perspective of statistical inference, this work advances the theory and methodology for statistical inference of large models. Specifically, it is theoretically proven that the proposed method permits the model size to increase with the sample size *n* at a rate of $O(n^{\zeta })$ for some $0 \le \zeta <1$, while still maintaining proper quantification of uncertainty in the model parameters. This result marks a significant improvement compared to the range $0\le \zeta < \frac{1}{2}$ required by the classical central limit theorem. Furthermore, this work provides a rigorous framework for quantifying the uncertainty of deep neural networks under the neural scaling law, representing a substantial contribution to the statistical understanding of large-scale neural network models.

**Supplementary Information:**

The online version contains supplementary material available at 10.1007/s11222-025-10624-8.

## Introduction

Causal inference is a fundamental problem in many disciplines such as medicine, econometrics, and social science. Formally, let $\{(y_1,{\varvec{x}}_1,t_1), (y_2,{\varvec{x}}_2,t_2),\ldots , (y_n,{\varvec{x}}_n,t_n)\}$ denote a set of observations drawn from the following data-generating equations:

1 $$\begin{aligned} y_i=c({\varvec{x}}_i) +\tau ({\varvec{x}}_i) t_i+\sigma z_i, \quad i=1,2,\ldots ,n, \end{aligned}$$

where $${\varvec{x}}_i \in \mathbb {R}^d$$ represents a vector of covariates of subject *i*, $$t_i \in \{0,1\}$$ represents the treatment assignment to subject *i*; $$c(\cdot )$$ represents the expected outcome of subject *i* if assigned to the control group (with $$t_i=0)$$, and $$\tau ({\varvec{x}}_i)$$ is the expected treatment effect of subject *i* if assigned to the treatment group (with $$t_i=1$$); $$\sigma >0$$ is the standard deviation, and $$z_i$$ represent a standardized random error that is not necessarily Gaussian. Under the potential outcome framework (Rubin 1974), each individual receives only one assignment of the treatment with $$t_i=0$$ or 1, but not both. The goal of causal inference is to make inference for the average treatment effect (ATE) or individual treatment effect (ITE).

The ATE is defined as

2 $$\begin{aligned} \tau _0=\mathbb {E}(\tau ({\varvec{x}}))=\int _{\mathcal {X}} \tau ({\varvec{x}}) dF({\varvec{x}}), \end{aligned}$$

where $\mathcal {X}$ denotes the sample space of ${\varvec{x}}$, and $F({\varvec{x}})$ denotes the cumulative distribution function of ${\varvec{x}}$. To estimate ATE, a variety of methods, including outcome regression, augmented/inverse probability weighting (AIPW/IPW) and matching, have been developed. See Imbens (2004) and Rosenbaum (2002) for overviews.

The ITE is often defined as the conditional average treatment effect (CATE):

3 $$\begin{aligned} \tau ({\varvec{x}})=\mathbb {E}(Y|T=1,{\varvec{x}})-\mathbb {E}(Y|T=0,{\varvec{x}}), \end{aligned}$$

see e.g., Shalit et al. (2017) and Lu et al. (2018). Recently, Lei and Candès (2021) proposed to make predictive inference of the ITE by quantifying the uncertainty of

4 $$\begin{aligned} \tilde{\tau }_i:=Y(T=1,{\varvec{x}}_i)-Y(T=0,{\varvec{x}}_i):=Y_i(1)-Y_i(0), \end{aligned}$$

where $Y_i(t_i)$ denotes the potential outcome of subject *i* with treatment assignment $t_i \in \{0,1\}$. Henceforth, we will call $\tilde{\tau }_i$ the predictive ITE.

It is known that ATE and ITE are identifiable if the conditions *‘strong ignorability’* and *‘overlapping’* are satisfied. The former means that, after accounting for observed covariates, the treatment assignment is independent of potential outcomes; and the latter ensures that every subject in the study has a positive probability of receiving either assignment, allowing for meaningful comparisons between treatment and control groups. Mathematically, the two conditions can be expressed as:where $T\in \{0,1\}$ represents the treatment assignment variable, and  denotes conditional independence. Together, they ensure that the causal effect can be correctly estimated without bias. See e.g. Guan and Yang (2019) for more discussions on this issue.

However, even under these assumptions, accurate inference for ATE and ITE can still be challenging. Specifically, the inference task can be complicated by unknown nonlinear forms of $c({\varvec{x}})$ and $\tau ({\varvec{x}})$. To address these issues, some authors have proposed to approximate them using a machine learning model, such as random forest (RF) (Breiman 2001), Bayesian additive regression trees (BART) (Chipman et al. 2010), and neural networks. Refer to e.g., Foster et al. (2011), Hill (2011), Shalit et al. (2017), Wager and Athey (2018), and Hahn et al. (2020) for the details. Unfortunately, these methods often yield point estimates for the ATE and ITE, while failing to correctly quantifying their uncertainty due to the complexity of the machine learning models. Quite recently, Lei and Candès (2021) proposed to quantify the uncertainty of the predictive ITE using the conformal inference method (Vovk et al. 2005; Shafer and Vovk 2008). This method provides coverage-guaranteed confidence intervals for the predictive ITE, but the intervals may become overly wide when the machine learning model is not consistently estimated. In short, while machine learning models, particularly neural networks, can effectively model complex, nonlinear functions such as $c(\cdot )$ and $\tau (\cdot )$ for causal inference, performing accurate uncertainty quantification with these models remains a significant challenge. This is because these models typically have a complex functional form and involve a large number of parameters.

In this paper, we propose to conduct causal inference using an extended fiducial inference (EFI) method (Liang et al. 2024), with the goal of addressing the uncertainty quantification issue associated with treatment effect estimation. EFI provides an innovative framework for inferring model uncertainty based solely on observed data, aligning with the goal of fiducial inference (Fisher 1935; Hannig 2009). Specifically, it aims to solve the data-generating equations by explicitly imputing the unobserved random errors and approximating the model parameters from the observations and imputed random errors using a neural network; it then infers the uncertainty of the model parameters based on the learned neural network function and the imputed random errors (see Section 2 for a brief review). To make the EFI method feasible for causal effect estimation with accurate uncertainty quantification, we extend the method in two key aspects:

(i) We approximate each of the unknown functions, $c({\varvec{x}})$ and $\tau ({\varvec{x}})$, by a deep neural network (DNN) model. The DNN possesses universal approximation capability (Hornik et al. 1989; Hornik 1991; Kidger and Lyons 2020), meaning it can approximate any continuous function to an arbitrary degree of accuracy, provided it is sufficiently wide and deep. This property makes the proposed method applicable to a wide range of data-generating processes.
(ii) We theoretically prove that the dimensions (i.e., the number of parameters) of the DNN models used to approximate $c({\varvec{x}})$ and $\tau ({\varvec{x}})$ are allowed to increase with the sample size *n* at a rate of $O(n^{\zeta })$ for some $0< \zeta <1$, while the uncertainty of the DNN models can still be correctly quantified. That is, we are able to correctly quantify the uncertainty of the causal effect although it has to be approximated using large models.

In this paper, we regard a model as ‘large’ if its dimension increases with *n* at a rate of $1/2\le \zeta < 1$. We note that part (ii) represents a significant theoretical innovation in statistical inference for large models. In the literature on this area, most efforts have focused on linear models, featuring techniques such as desparsified Lasso (Javanmard and Montanari 2014; van de Geer et al. 2014; Zhang and Zhang 2014), post-selection inference (Lee et al. 2016), and Markov neighborhood regression (Liang et al. 2022a). For nonlinear models, the research landscape appears to be more scattered. Portnoy (1986, 1988) showed that for independently and identically distributed (i.i.d) random vectors with the dimension *p* increasing with the sample size *n*, the central limit theorem (CLT) holds if $p=O(n^{\zeta })$ for some $0\le \zeta <1/2$. It is worth noting that Bayesian methods, despite being sampling-based, do not permit the dimension of the true model to increase with *n* at a higher rate. For example, even in the case of generalized linear models, to ensure the posterior consistency, the dimension of the true model is only allowed to increase with *n* at a rate $0 \le \zeta < 1/4$ (see Theorem 2 and Remark 2 of Jiang (2007)). Under its current theoretical framework developed by Liang et al. (2024), EFI can only be applied to make inference for the models whose dimension is fixed or increases with *n* at a very low rate. This paper extends the theoretical framework of EFI further, establishing its applicability for statistical inference of large models.

It is worth noting that a DNN model with size $p=O(n^{\zeta })$, where $\zeta$ is close to (but less than) 1, has been shown to be sufficiently large for approximating many data generation processes. This is supported by the theory established in Sun et al. (2022) and Farrell et al. (2021). In Sun et al. (2022), it is shown that, as $n \rightarrow \infty$, a sparse DNN model of this size can provide accurate approximations for multiple classes of functions, such as bounded $\alpha$-Hölder smooth functions (Schmidt-Hieber 2020), piecewise smooth functions with fixed input dimensions (Petersen and Voigtlaender 2018), and functions representable by an affine system (Bolcskei et al. 2019). Similar results have also been obtained in Farrell et al. (2021), where it is shown that a multi-layer perceptron (MLP) with this model size and the ReLU activation function can provide an accurate approximation to the functions that lie in a Sobolev ball with certain smoothness. The approximation capability of DNNs of this size has also been empirically validated by Hestness et al. (2017), where a neural scaling law of $p =O(n^{\zeta })$ with $0.5 \le \zeta <1$ was identified through extensive studies across various model architectures in machine translation, language modeling, image processing, and speech recognition.

To highlight the strength of EFI in uncertainty quantification and to facilitate comparison with the conformal inference method, this study focuses on inference for predictive ITEs, although the proposed method can also be extended to ATE and CATE. Our numerical results demonstrate the superiority of the proposed method over the conformal inference method.

The remaining part of this paper is organized as follows. Section 2 provides a brief review of the EFI method. Section 3 extends EFI to statistical inference for large statistical models. Section 4 provides an illustrative example for EFI. Section 5 applies the proposed method to statistical inference for predictive ITEs, with both simulated and real data examples. Section 6 concludes the paper with a brief discussion.

## A Brief Review of the EFI Method

While fiducial inference was widely considered as a big blunder by R.A. Fisher, the goal he initially set —inferring the uncertainty of model parameters on the basis of observations — has been continually pursued by many statisticians, see e.g. Zabell (1992); Hannig (2009); Hannig et al. (2016); Murph et al. (2022), and Martin (2023). To this end, Liang et al. (2024) developed the EFI method based on the fundamental concept of structural inference (Fraser 1966, 1968). Consider a regression model:

5 $$\begin{aligned} Y=f({\varvec{X}},Z,{\varvec{\theta }}), \end{aligned}$$

where $Y\in \mathbb {R}$ and ${\varvec{X}}\in \mathbb {R}^{d}$ represent the response and explanatory variables, respectively; ${\varvec{\theta }}\in \mathbb {R}^p$ represents the vector of parameters; and $Z\in \mathbb {R}$ represents a scaled random error following a known distribution $\pi _0(\cdot )$. For the model (1), the treatment assignment *T* should be included as a part of ${\varvec{X}}$.

Suppose that a random sample of size *n* has been collected from the model, denoted by $\{(y_1,{\varvec{x}}_1), (y_2,{\varvec{x}}_2),\ldots ,(y_n,{\varvec{x}}_n)\}$. In the point of view of structural inference (Fraser 1966, 1968), they can be expressed in the data generating equations as follow:

6 $$\begin{aligned} y_i=f({\varvec{x}}_i,z_i,{\varvec{\theta }}), \quad i=1,2,\ldots ,n. \end{aligned}$$

This system of equations consists of $n+p$ unknowns, namely, $\{{\varvec{\theta }}, z_1, z_2, \ldots , z_n \}$, while there are only *n* equations. Therefore, the values of ${\varvec{\theta }}$ cannot be uniquely determined by the data-generating equations, and this lack of uniqueness of unknowns introduces uncertainty in ${\varvec{\theta }}$.

Let ${\varvec{Z}}_n=\{z_1,z_2,\ldots ,z_n\}$ denote the unobservable random errors, which are also called latent variables in EFI. Let $G(\cdot )$ denote an inverse function/mapping for the parameter ${\varvec{\theta }}$, i.e.,

7 $$\begin{aligned} {\varvec{\theta }}=G({\varvec{Y}}_n,{\varvec{X}}_n,{\varvec{Z}}_n). \end{aligned}$$

It is worth noting that the inverse function is generally non-unique. For example, it can be constructed by solving any *p* equations in (6) for ${\varvec{\theta }}$. As noted by Liang et al. (2024), this non-uniqueness of inverse function mirrors the flexibility of frequentist methods, where different estimators of ${\varvec{\theta }}$ can be designed for different purposes.

**Fig. 1:**
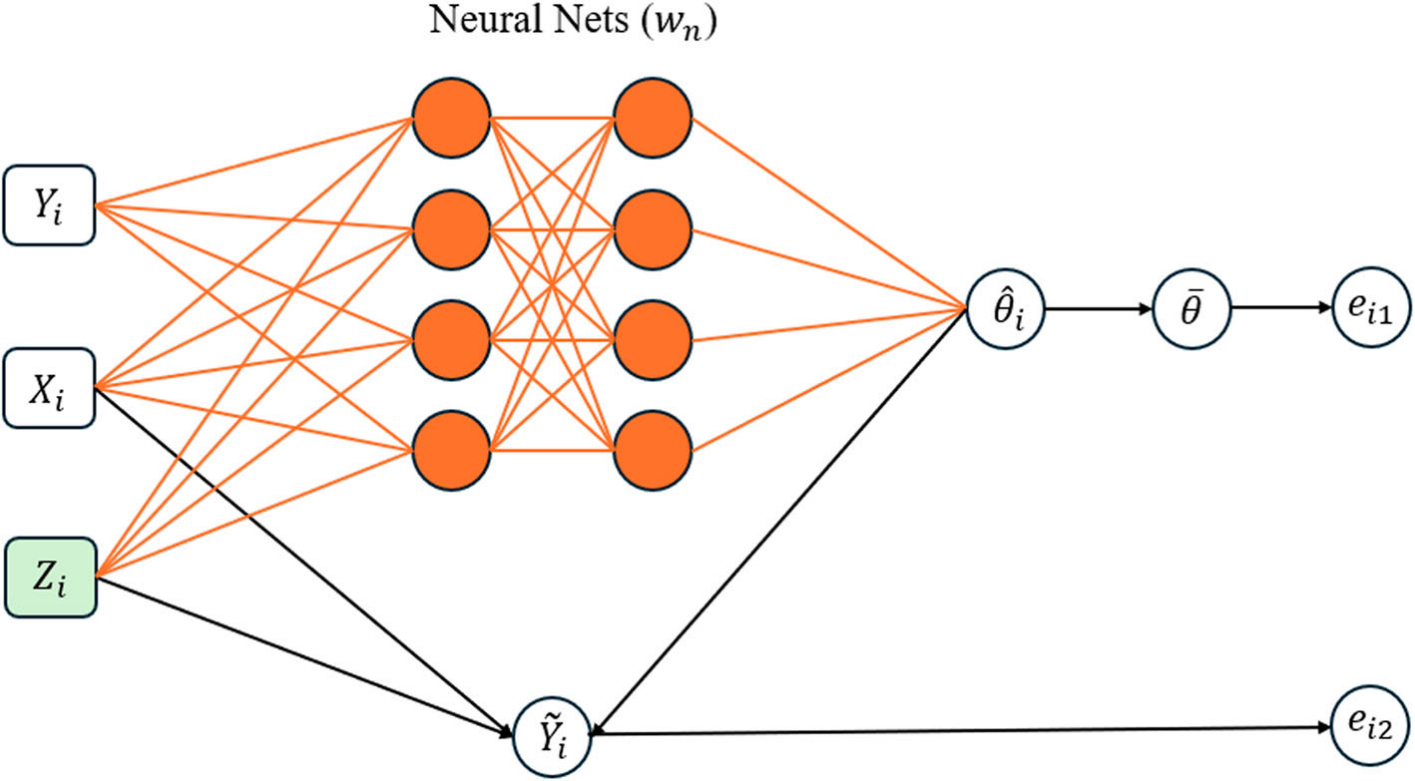
Illustration of the EFI network (Liang et al. 2024), where the orange nodes and orange links form a DNN (parameterized by the weights ${\varvec{w}}_n$, with the subscript *n* indicating its dependence on the training sample size *n*), the green node represents latent variable to impute, and the black lines represent deterministic functions

As a general method, Liang et al. (2024) proposed to approximate the inverse function $G(\cdot )$ using a sparse DNN, see Figure 1 for illustration. They also introduced an adaptive stochastic gradient Langevin dynamics (SGLD) algorithm, which facilitates the simultaneous training of the sparse DNN and simulation of the latent variables ${\varvec{z}}$. This is briefly described as follows.

Let $\hat{{\varvec{\theta }}}_i:=\hat{g}(y_i,{\varvec{x}}_i,z_i,{\varvec{w}}_n)$ denote the DNN prediction function parameterized by the weights ${\varvec{w}}_n$ in the EFI network, and let

8 $$\begin{aligned} \bar{{\varvec{\theta }}}:=\frac{1}{n} \sum _{i=1}^n \hat{{\varvec{\theta }}}_i=\frac{1}{n} \sum _{i=1}^n \hat{g}(y_i,{\varvec{x}}_i,z_i,{\varvec{w}}_n), \end{aligned}$$

which serves as an estimator of $G(\cdot )$. The EFI network has two output nodes defined, respectively, by

9 $$\begin{aligned} e_{i1} :=\Vert \hat{{\varvec{\theta }}}_i-\bar{{\varvec{\theta }}}\Vert ^2, \quad e_{i2} :=d(y_i,\tilde{y}_i):=d(y_i,{\varvec{x}}_i, z_i, \bar{{\varvec{\theta }}}), \end{aligned}$$

where $\tilde{y}_i=f({\varvec{x}}_i,z_i,\bar{{\varvec{\theta }}})$, $f(\cdot )$ is as specified in (6), and $d(\cdot )$ is a function that measures the difference between $y_i$ and $\tilde{y}_i$. For example, for a normal linear/nonlinear regression, it can be defined as

10 $$\begin{aligned} d(y_i,{\varvec{x}}_i,z_i,\bar{{\varvec{\theta }}})=\Vert y_i-f({\varvec{x}}_i,z_i,\bar{{\varvec{\theta }}})\Vert ^2. \end{aligned}$$

For logistic regression, it is defined as a squared ReLU function, see Liang et al. (2024) for the details. Furthermore, EFI defines an energy function as follows:

11 $$\begin{aligned} U_n({\varvec{Y}}_n,{\varvec{X}}_n,{\varvec{Z}}_n,{\varvec{w}}_n) = \sum _{i=1}^n d(y_i,{\varvec{x}}_i,z_i,\bar{{\varvec{\theta }}}) + \eta \sum _{i=1}^n\Vert \hat{{\varvec{\theta }}}_i- \bar{{\varvec{\theta }}} \Vert ^2, \end{aligned}$$

for some regularization coefficient $\eta >0$, where first term measures the fitting error of the model as implied by equation (10), and the second term regularizes the variation of $\hat{{\varvec{\theta }}}_i$, ensuring that the neural network forms a proper estimator of the inverse function. Given this energy function, we define the likelihood function as

12 $$\begin{aligned} \pi _{\epsilon }({\varvec{Y}}_n|{\varvec{X}}_n,{\varvec{Z}}_n,{\varvec{w}}_n) \propto e^{- U_n({\varvec{Y}}_n,{\varvec{X}}_n,{\varvec{Z}}_n,{\varvec{w}}_n)/\epsilon }, \end{aligned}$$

for some constant $\epsilon$ close to 0. As discussed in Liang et al. (2024), the choice of $\eta$ does not have much affect on the performance of EFI as long as $\epsilon$ is sufficiently small.

Subsequently, the posterior of ${\varvec{w}}_n$ is given by

13 $$\begin{aligned} \begin{aligned} \pi _{\epsilon }({\varvec{w}}_n|{\varvec{X}}_n,{\varvec{Y}}_n,{\varvec{Z}}_n)&\propto \pi ({\varvec{w}}_n) e^{-U_n({\varvec{Y}}_n,{\varvec{X}}_n,{\varvec{Z}}_n,{\varvec{w}}_n)/\epsilon }, \end{aligned} \end{aligned}$$

where $\pi ({\varvec{w}}_n)$ denotes the prior of ${\varvec{w}}_n$; and the predictive distribution of ${\varvec{Z}}_n$ is given by

14 $$\begin{aligned} \begin{aligned} \pi _{\epsilon }({\varvec{Z}}_n|{\varvec{X}}_n,{\varvec{Y}}_n,{\varvec{w}}_n)&\propto \pi _0^{\otimes n}({\varvec{Z}}_n) e^{-U_n({\varvec{Y}}_n,{\varvec{X}}_n,{\varvec{Z}}_n,{\varvec{w}}_n)/\epsilon }. \end{aligned} \end{aligned}$$

In EFI, ${\varvec{w}}_n$ is estimated through maximizing the posterior $\pi _{\epsilon }({\varvec{w}}_n|{\varvec{X}}_n,{\varvec{Y}}_n)$ given the observations $\{{\varvec{X}}_n,{\varvec{Y}}_n \}$. By the Bayesian version of Fisher’s identity (Song et al. 2020), the gradient equation $\nabla _{{\varvec{w}}_n} \log \pi _{\epsilon }({\varvec{w}}_n|{\varvec{X}}_n,{\varvec{Y}}_n)$ $=0$ can be re-expressed as

15 $$\begin{aligned} \nabla _{{\varvec{w}}_n} \log \pi _{\epsilon }({\varvec{w}}_n|{\varvec{X}}_n,{\varvec{Y}}_n)\!=\! &  \int \!\nabla _{{\varvec{w}}_n} \log \pi _{\epsilon }({\varvec{w}}_n|{\varvec{X}}_n,{\varvec{Y}}_n,{\varvec{Z}}_n) \pi _{\epsilon }\nonumber \\ &  ({\varvec{Z}}_n|{\varvec{X}}_n,{\varvec{Y}}_n,{\varvec{w}}_n) d{\varvec{w}}_n=0, \end{aligned}$$

which can be solved using an adaptive stochastic gradient MCMC algorithm (Liang et al. 2022b; Deng et al. 2019). The algorithm works by iterating between two steps:

- *Latent variable sampling*: draw ${\varvec{Z}}_n^{(k+1)}$ according to a Markov transition kernel that leaves $\pi _{\epsilon }({\varvec{z}}|{\varvec{X}}_n,{\varvec{Y}}_n,{\varvec{w}}_n^{(k)})$ to be invariant;
- *Parameter updating*: update ${\varvec{w}}_n^{(k)}$ toward the maximum of $\log \pi _{\epsilon }({\varvec{w}}_n|{\varvec{X}}_n,{\varvec{Y}}_n,{\varvec{Z}}_n)$ using stochastic approximation (Robbins and Monro 1951), based on the sample ${\varvec{Z}}_n^{(k+1)}$.

See Algorithm 1 for the pseudo-code. This algorithm is termed “adaptive” because the transition kernel in the latent variable sampling step changes with the working parameter estimate of ${\varvec{w}}_n$. The parameter updating step can be implemented using mini-batch SGD, and the latent variable sampling step can be executed in parallel for each observation $(y_i,{\varvec{x}}_i)$. Hence, the algorithm is scalable with respect to large datasets.

**Algorithm 1:**
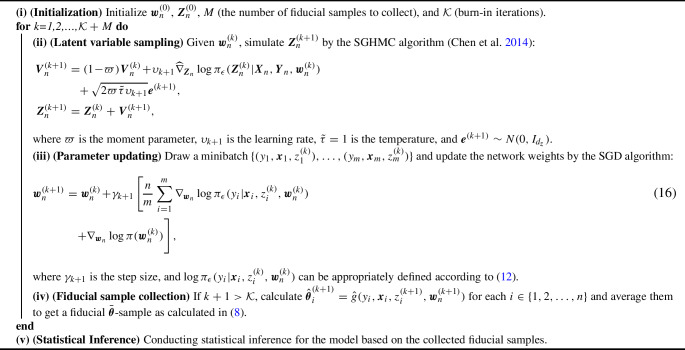
Adaptive SGHMC for Extended Fiducial Inference

Under mild conditions for adaptive stochastic gradient MCMC algorithms (Deng et al. 2019; Liang et al. 2022b), it is shown in Liang et al. (2024) that

17 $$\begin{aligned} \Vert {\varvec{w}}_n^{(k)} -{\varvec{w}}_n^* \Vert {\mathop {\rightarrow }\limits ^{p}} 0, \quad \text{ as } k\rightarrow \infty , \end{aligned}$$

where ${\varvec{w}}_n^*$ denotes a solution to equation (15) and ${\mathop {\rightarrow }\limits ^{p}}$ denotes convergence in probability, and that

18 $$\begin{aligned} {\varvec{Z}}_n^{(k)} {\mathop {\rightsquigarrow }\limits ^{d}} \pi _{\epsilon }({\varvec{Z}}_n|{\varvec{X}}_n,{\varvec{Y}}_n,{\varvec{w}}_n^*), \quad \text{ as } k \rightarrow \infty , \end{aligned}$$

in 2-Wasserstein distance, where ${\mathop {\rightsquigarrow }\limits ^{d}}$ denotes weak convergence.

To study the limit of (18) as $\epsilon$ decays to 0, i.e.,

$$\begin{aligned} p_n^*({\varvec{z}}|{\varvec{Y}}_n,{\varvec{X}}_n,{\varvec{w}}_n^*)= \lim _{\epsilon \downarrow 0} \pi _{\epsilon }({\varvec{Z}}_n|{\varvec{X}}_n,{\varvec{Y}}_n,{\varvec{w}}_n^*), \end{aligned}$$

where $p_n^*({\varvec{z}}|{\varvec{Y}}_n,{\varvec{X}}_n,{\varvec{w}}_n^*)$ is referred to as the extended fiducial density (EFD) of ${\varvec{Z}}_n$ learned in EFI, it is necessary for ${\varvec{w}}_n^*$ to be a consistent estimator of ${\varvec{w}}_*$, the parameters of the underlying true EFI network. To ensure this consistency, Liang et al. (2024) impose some conditions on the structure of the DNN and the prior distribution $\pi ({\varvec{w}}_n)$. Specifically, they assume that ${\varvec{w}}_n$ takes values in a compact space $\mathcal {W}$; $\pi ({\varvec{w}}_n)$ is a truncated mixture Gaussian distribution on $\mathcal {W}$; and the DNN structure satisfies certain constraints given in Sun et al. (2022), e.g., the width of the output layer (i.e., the dimension of ${\varvec{\theta }}$) is fixed or grows very slowly with *n*. They then justify the consistency of ${\varvec{w}}_n^*$ based on the sparse deep learning theory developed in Sun et al. (2022). The consistency of ${\varvec{w}}_n^*$ further implies that

$$\begin{aligned} G^*({\varvec{Y}}_n,{\varvec{X}}_n,{\varvec{Z}}_n)= \frac{1}{n} \sum _{i=1}^n \hat{g}(y_i,{\varvec{x}}_i,z_i,{\varvec{w}}_n^*), \end{aligned}$$

serves as a consistent estimator for the inverse function/mapping ${\varvec{\theta }}=G({\varvec{Y}}_n,{\varvec{X}}_n,{\varvec{Z}}_n)$.

By Theorem 3.2 in Liang et al. (2024), for the target model (1), which is a noise-additive model, the EFD of ${\varvec{Z}}_n$ is invariant to the choice of the inverse function, provided that $d(\cdot )$ is specified as in (10) in defining the energy function. Further, by Lemma 4.2 in Liang et al. (2024), $p_n^*({\varvec{z}}|{\varvec{Y}}_n,{\varvec{X}}_m,{\varvec{w}}_n^*)$ is given by

19 $$\begin{aligned} \frac{dP_n^*({\varvec{z}}|{\varvec{X}}_n,{\varvec{Y}}_n,{\varvec{w}}_n^*)}{d\nu }= \frac{\pi _0^{\otimes n}({\varvec{z}})}{\int _{\mathcal {Z}_n} \pi _0^{\otimes n}({\varvec{z}}) d \nu }, \end{aligned}$$

where $P_n^*({\varvec{z}}|{\varvec{X}}_n,{\varvec{Y}}_n,{\varvec{w}}_n^*)$ represents the cumulative distribution function (CDF) corresponding to $p_n^*({\varvec{z}}|{\varvec{X}}_n,{\varvec{Y}}_n,{\varvec{w}}_n^*)$; $\mathcal {Z}_n=\{{\varvec{z}}: U_n({\varvec{Y}}_n,{\varvec{X}}_n,{\varvec{Z}}_n, {\varvec{w}}_n^*)=0\}$ represents the zero-energy set, which forms a manifold in the space $\mathbb {R}^n$; and $\nu$ is the sum of intrinsic measures on the *p*-dimensional manifold in $\mathcal {Z}_n$. That is, under the consistency of ${\varvec{w}}_n^*$, $p_n^*({\varvec{z}}|{\varvec{X}}_n,{\varvec{Y}}_n,{\varvec{w}}_n^*)$ is reduced to a truncated density function of $\pi _0^{\otimes n}({\varvec{z}})$ on the manifold $\mathcal {Z}_n$, while $\mathcal {Z}_n$ itself is also invariant to the choice of the inverse function as shown in Lemma 3.1 of Liang et al. (2024). In other words, for the model (1), the EFD of ${\varvec{Z}}_n$ is asymptotically invariant to the inverse function we learned given its consistency.

Let $\Theta :=\{{\varvec{\theta }}\in \mathbb {R}^p: {\varvec{\theta }}=G^*({\varvec{Y}}_n,{\varvec{X}}_n,{\varvec{z}}), {\varvec{z}}\in \mathcal {Z}_n\}$ denote the parameter space of the target model, which represents the set of all possible values of ${\varvec{\theta }}$ that $G^*(\cdot )$ takes when ${\varvec{z}}$ runs over $\mathcal {Z}_n$. Then, for any function $b({\varvec{\theta }})$ of interest, its EFD $\mu _n^*(\cdot |{\varvec{Y}}_n,{\varvec{X}}_n)$ associated with $G^*(\cdot )$ is given by

20 $$\begin{aligned} \begin{aligned}&\mu _n^*(B|{\varvec{Y}}_n,{\varvec{X}}_n) \\&\quad =\int _{\mathcal {Z}_n(B)} d P_n^*({\varvec{z}}|{\varvec{Y}}_n,{\varvec{X}}_n,{\varvec{w}}_n^*), \quad \text{ for } \text{ any } \text{ measurable } \text{ set } B \subset \Theta , \end{aligned}\nonumber \\ \end{aligned}$$

where $\mathcal {Z}_n(B)=\{{\varvec{z}}\in \mathcal {Z}_n: b(G^*({\varvec{Y}}_n,{\varvec{X}}_n,{\varvec{z}})) \in B\}$. The EFD provides an uncertainty measure for $b({\varvec{\theta }})$. Practically, the EFD of $b({\varvec{\theta }})$ can be constructed based on the samples $\{b(\bar{{\varvec{\theta }}}_1), b(\bar{{\varvec{\theta }}}_2), \ldots , b(\bar{{\varvec{\theta }}}_M)\}$, where $\{\bar{{\varvec{\theta }}}_1, \bar{{\varvec{\theta }}}_2, \ldots , \bar{{\varvec{\theta }}}_M\}$ denotes the fiducial $\bar{{\varvec{\theta }}}$-samples collected at step (iv) of Algorithm 1.

Finally, we note that, as discussed in Liang et al. (2024), the invariance property of $\mathcal {Z}_n$ is not crucial to the validity of EFI, although it does enhance the robustness of the inference. Additionally, for a neural network model, its parameters are only unique up to certain loss-invariant transformations, such as reordering hidden neurons within the same hidden layer or simultaneously altering the sign or scale of certain connection weights, see Sun et al. (2022) for discussions. Therefore, in EFI, the consistency of ${\varvec{w}}_n^*$ refers to its consistency with respect to one of the equivalent solutions to (15), while mathematically ${\varvec{w}}_n^*$ can still be treated as unique. Refer to Section §1.1 (of the supplement) for more discussions on this issue.

## EFI for Large Models

In this section, we first establish the consistence of the inverse function/mapping learned in EFI for large models, and then discuss its application for uncertainty quantification of deep neural networks.

### Consistency of Inverse Mapping Learned in EFI for Large Models

It is important to note that the sparse deep learning theory of Sun et al. (2022) is developed under the general constraint $dim({\varvec{w}}_n)=O(n^{1-\delta })$ for some $0<\delta <1$, which restricts the dimension of the output layer of the DNN model to be fixed or grows very slowly with the sample size *n*. Therefore, under its current theoretical framework, EFI can only be applied to the models for which the dimension is fixed or increases very slowly with *n*.

To extend EFI to large models, where the dimension of ${\varvec{\theta }}$ can grow with *n* at a rate of $O(n^{\zeta })$, particularly for $1/2\le \zeta <1$, we provide a new proof for the consistency of $G^*({\varvec{Y}}_n,{\varvec{X}}_n,{\varvec{Z}}_n)$ based on the theory of stochastic deep learning (Liang et al. 2022b). Specifically, we establish the following theorem, where the output layer width of the DNN in the EFI network is set to match the dimension of ${\varvec{\theta }}$. The proof is lengthy and provided in the supplement.

#### Theorem 3.1

Suppose Assumptions 1-6 hold (see the supplement), $\epsilon$ is sufficiently small, and

21 $$\begin{aligned} \sum _{l=1}^H d_l \prec n, \end{aligned}$$

where $d_l$ denotes the width of layer *l*, $d_H=dim({\varvec{\theta }})$, and *H* denotes the depth of the DNN in the EFI network. Then $G^*({\varvec{Y}}_n,{\varvec{X}}_n,{\varvec{Z}}_n)= \frac{1}{n} \sum _{i=1}^n \hat{g}(y_i,{\varvec{x}}_i,z_i,{\varvec{w}}_n^*)$ constitutes a consistent estimator of the inverse function.

As implied by (21), we have $d_l \prec n$ holds for each layer $l=1,2,\ldots ,H$. We call such a neural network a narrow DNN. For narrow DNNs, by the existing theory, see e.g., Kidger and Lyons (2020), Park et al. (2020), and Kim et al. (2023), the universal approximation can be achieved with a minimum hidden layer width of $\max \{d_0+1, d_H\}$, where $d_0$ and $d_H$ represent the widths of the input and output layers, respectively. Hence, (21) implies that EFI can be applied to statistical inference for a large model of dimension

$$\begin{aligned} dim({\varvec{\theta }})=d_H =O(n^{\zeta }), \quad 0 \le \zeta <1, \end{aligned}$$

under the narrow DNN setting with the depth $H=O(n^{\beta })$ for some $0<\beta <1-\zeta$. Here, Without loss of generality, we assume $d_0 \preceq d_H$. For such a DNN, the total dimension of ${\varvec{w}}_n$:

$$\begin{aligned} dim({\varvec{w}}_n)=\sum _{i=1}^H d_i (d_{i-1}+1) =O(n^{2\zeta +\beta }), \end{aligned}$$

can be much greater than *n*, where ‘1’ represents the bias parameter of each neuron at the hidden and output layers. Specifically, we can have $dim({\varvec{w}}_n) \succ n$ with appropriate choices of $\zeta$ and $\beta$. However, leveraging the asymptotic equivalence between the DNN and an auxiliary stochastic neural network (StoNet) (Liang et al. 2022b), we can still prove that the resulting estimator of ${\varvec{\theta }}$ is consistent, see the supplement for the detail.

Regarding this extension of the EFI method for statistical inference of large models, we have an additional remark:

#### Remark 1

In this paper, we impose a mixture Gaussian prior on ${\varvec{w}}_n$ to ensure the consistency of ${\varvec{w}}_n^*$ and, consequently, the consistency of the inverse mapping $G^*({\varvec{Y}}_n,{\varvec{X}}_n,{\varvec{Z}}_n)$. However, this Bayesian treatment of ${\varvec{w}}_n$ is not strictly necessary, although it introduces sparsity that improves the efficiency of EFI. For the narrow DNN, the consistency of the ${\varvec{w}}_n$ estimator can also be established under the frequentist framework by leveraging the asymptotic equivalence between the DNN and the auxiliary StoNet, using the same technique introduced in the supplement (see Section §1.2). In this narrow and deep setting, each of the regressions formed by the StoNet is low-dimensional (with $d_l \prec n$), making the Bayesian treatment of ${\varvec{w}}_n$ unnecessary while still achieving a consistent estimator of ${\varvec{w}}_n$.

### Double-NN Method

Suppose a DNN is used for modeling the data, i.e., approximating the function $f(\cdot )$ in (5). By Sun et al. (2022) and Farrell et al. (2021), a DNN of size $O(n^{\zeta })$ for some $0<\zeta <1$ has been large enough for approximating many classes of functions. Therefore, EFI can be used for making inference for such a DNN model. In this case, EFI involves two neural networks, one is for modeling the data, which is called the ‘data modeling network’ and parameterized by ${\varvec{\theta }}$; and the other one is for approximating the inverse function, which is called the ‘inverse mapping network’ and parameterized by ${\varvec{w}}_n$. Therefore, the proposed method is coined as ‘double-NN’. Note that during the EFI training process, only the parameters ${\varvec{w}}_n$ of the inverse mapping network are updated in equation (16) of Algorithm 1. The parameters of the data modeling network are subsequently updated in response to the adjustment of ${\varvec{w}}_n$, based on the formula given in (8).

In our theoretical study for the double-NN method, we actually assume that the true data-generating model $Y=f({\varvec{X}},Z,{\varvec{\theta }})$ is a neural network, thereby omitting the approximation error of the data modeling network, based on its universal approximation capability. In practice, we have observed that the double-NN method is robust to this approximation error. Specifically, even when the true model is not a neural network, EFI can still recover the true random errors with high accuracy and achieve the zero-energy solution as $n\rightarrow \infty$ and $\epsilon \rightarrow 0$. A further theoretical exploration of this phenomenon would be of interest.

As mentioned previously, for a neural network model, its parameters are only unique up to certain loss-invariant transformations. As the training sample size *n* becomes large, we expect that the optimizers $\hat{{\varvec{\theta }}}:=\arg \max _{{\varvec{\theta }}} \pi _{\epsilon }({\varvec{Z}}_n|{\varvec{X}}_n,{\varvec{Y}}_n,{\varvec{\theta }})$ are all equivalent. Thus, in this paper, the consistency of $\hat{{\varvec{\theta }}}$ refers to its consistency with respect to one of the equivalent global optimizers, while mathematically $\hat{{\varvec{\theta }}}$ can still be treated as unique. A similar issue occurs to the parameters of the inverse mapping network, as discussed in Section §1.1 of the supplement.

## An Illustrative Example for EFI

To illustrate how EFI works for statistical inference problems, we consider a linear regression example:

22 $$\begin{aligned} y_i=\tau T_i +\mu +{\varvec{x}}_i^{\top } {\varvec{\beta }}+\sigma z_i, \quad i=1,2,\ldots ,n, \end{aligned}$$

where $T_i \in \{0,1\}$ is a binary variable indicating the treatment assignment, $\tau$ is the treatment effect, ${\varvec{x}}_i \in \mathbb {R}^d$ are confounders/covariates, $z_i\sim N(0,1)$ is the standardized random noise, and ${\varvec{\beta }}\in \mathbb {R}^d$ and $\sigma \in \mathbb {R}_+$ are unknown parameters. For this example, $\tau$ represents the ATE as well as the CATE, due to its independence of the covariates ${\varvec{x}}$. In the simulation study, we set $\tau =1$, $\mu =1$, $d=4$, and ${\varvec{\beta }}=(-1,1,-1,1)^{\top }$; generate ${\varvec{x}}_i \sim N(0,I_d)$; and generate the treatment variable via a logistic regression:

23 $$\begin{aligned} P(T_i=1)=\frac{1}{1+\exp \{-\nu -{\varvec{\xi }}^{\top } {\varvec{x}}_i \}}, \end{aligned}$$

where $\nu =1$ and ${\varvec{\xi }}=(-1,1,-1,1)^{\top }$. We consider three different cases with the sample size $n=250$, 500 and 1000, respectively. For each case, we generate 100 datasets.

Statistical inference for the parameters in the model (22) can be made with EFI under its standard framework. Let ${\varvec{\theta }}=(\tau ,\mu ,{\varvec{\beta }}^{\top },\log \sigma )^{\top }$ be the parameter vector. EFI approximates the inverse function ${\varvec{\theta }}=g(y,T,{\varvec{x}},z)$ by a DNN, for which $(y,T,{\varvec{x}},z)$ serves as input variables and ${\varvec{\theta }}$ as output variables. The results are summarized in Table 1.

For comparison, a variety of methods, including Unadj (Imbens and Rubin 2015), inverse probability weighting (IPW) (Rosenbaum 1987), double-robust (DR) (Robins et al. 1994; Bang and Robins 2005), and BART (Hill 2011), have been applied to this example. These methods fall into distinct categories. The Unadj is straightforward, estimating the ATE by calculating the difference between the treatment and control groups, i.e., $\hat{\tau }=\frac{1}{n_t} \sum _{i=1}^{n_t} Y_i(1)- \frac{1}{n_c} \sum _{i=1}^{n_c} Y_i(0)$, where the effect of confounders is not adjusted. Both IPW and DR are widely used ATE estimation methods, which adjust the effect of confounders based on propensity scores. They both are implemented using the R package *drgee* (Zetterqvist and Sjölander 2015). The BART employs Bayesian additive regression trees to learn the outcome function, which naturally accommodates heterogeneous treatment effects as well as nonlinearity of the outcome function. It is implemented using the R package *bartcause* (Dorie and Hill 2020).

**Table 1:** Comparison of EFI with various ATE estimation methods, where “coverage” refers to the averaged coverage rate of $\tau$, “length” refers to the averaged width of confidence intervals, and the number in the parentheses refers to the standard deviation of the averaged width. The averages and standard deviations were calculated based on 100 datasets

	$n=250$		$n=500$		$n=1000$	
Method	coverage	length	coverage	length	coverage	length
Unadj	0.95	1.161(0.066)	0.93	0.822(0.032)	0.97	0.424(0.017)
BART	0.99	0.857(0.070)	0.98	0.611(0.047)	0.96	0.428(0.024)
IPW	0.90	0.710(0.157)	0.92	0.560(0.141)	0.92	0.417(0.101)
DR	0.96	0.652(0.058)	0.93	0.465(0.033)	0.94	0.331(0.017)
EFI	0.95	0.647(0.033)	0.95	0.438(0.021)	0.95	0.338(0.012)

The comparison indicates that EFI performs very well for this standard ATE estimation problem. Specifically, EFI generates confidence intervals of nearly the same length as DR, but with more accurate coverage rates. This is remarkable, as DR has often been considered as the golden standard for ATE estimation and is consistent if either the outcome or propensity score models is correctly specified, and locally efficient if both are correctly specified. Furthermore, EFI produces much shorter confidence intervals compared to Unadj, IPW, and BART, while maintaining more accurate coverage rates.

We attribute the superior performance of EFI on this example to its fidelity in parameter estimation, an attractive property of EFI as discussed in Liang et al. (2024). As implied by (14), EFI essentially estimates ${\varvec{\theta }}$ by maximizing the predictive likelihood function $\pi _{\epsilon }({\varvec{Z}}_n|{\varvec{X}}_n,{\varvec{Y}}_n,{\varvec{\theta }})\propto \pi _0^{\otimes n}({\varvec{Z}}_n) e^{- U_n({\varvec{Y}}_n,{\varvec{X}}_n,{\varvec{Z}}_n, {\varvec{w}})/\epsilon }$, which balances the likelihood of ${\varvec{Z}}_n$ and the model fitting errors coded in $U_n(\cdot )$. In contrast, the maximum likelihood estimation (MLE) method sets $\hat{{\varvec{\theta }}}_{MLE}= \arg \max _{{\varvec{\theta }}}\pi _0^{\otimes n}({\varvec{Z}}_n)$, where ${\varvec{Z}}_n$ is expressed as a function of $({\varvec{Y}}_n,{\varvec{X}}_n,{\varvec{\theta }})$. In general, MLE is inclined to be influenced by the outliers and deviations of covariates especially when the sample size is not sufficiently large. It is important to note that the MLE serves as the core for all the IPW, DR and BART methods in estimating the outcome and propensity score models. For this reason, various adjustments for confounding and heterogeneous treatment effects have been developed in the literature.

Compared to the existing causal inference methods, EFI works as a solver for the data-generating equation (as $\epsilon \downarrow 0$), providing a coherent way to address the confounding and heterogeneous treatment effects and resulting in faithful estimates for the model parameters and their uncertainty as well. This example illustrates the performance of EFI in ATE estimation when confounders are present, while the examples in the next section showcase the performance of EFI in dealing with heterogeneous treatment effects via DNN modeling. Extensive comparisons with BART and other nonparametric modeling methods are also presented.

In this example, we omit the estimation of the propensity score model. As discussed in Section 6, the proposed method can be extended by including an additional DNN to approximate the propensity score, enabling the use of inverse probability weighting for ATE estimation. However, the ATE estimation is not the focus of this work.

## Causal Inference for Individual Treatment Effects

This section demonstrates how EFI can be used to perform statistical inference of the predictive ITE for the data-generating model (1). Let ${\varvec{\theta }}_c$ denote the vector of parameters for modeling the function $c({\varvec{x}})$, let ${\varvec{\theta }}_{\tau }$ denote the vector of parameters for modeling the function $\tau ({\varvec{x}})$, and let ${\varvec{\theta }}=\{{\varvec{\theta }}_c,{\varvec{\theta }}_{\tau },\log (\sigma )\}$ denote the whole set of parameters for the model (1). We model the inverse function ${\varvec{\theta }}=g(y,T,{\varvec{x}},z)$ by a DNN. Also, we can model each of the functions $c({\varvec{x}})$ and $\tau ({\varvec{x}})$ by a DNN if their functional forms are unknown. For convenience, we refer to the DNN for modeling $c({\varvec{x}})$ as ‘*c*-network’ and that for modeling $\tau ({\varvec{x}})$ as ‘$\tau$-network’, and ${\varvec{\theta }}_c$ and ${\varvec{\theta }}_{\tau }$ represent their weights, respectively. As mentioned previously, we can restrict the sizes of the *c*-network and $\tau$-network to the order of $O(n^{\tilde{\zeta }})$ for some $0<\tilde{\zeta }<1$.

Note that in solving the data generating equations (1), the proposed method involves two types of neural networks: one for modeling causal effects and the other for approximating the inverse function ${\varvec{\theta }}=g(y,T,{\varvec{x}},z)$. While we still refer to the proposed method as ‘Double-NN’, it actually involves three DNNs.

### ITE prediction intervals

Assume the training set consists of $n_{train}$ subjects, and the test set consists of $n_{test}$ subjects. The subjects in the test set can be grouped into three categories: (i) $\{({\varvec{x}}_i,0,Y_i(1),Y_i^{obs}(0))$, $i\in \mathcal {I}_c\}$, where the responses under the control are observed; (ii) $\{({\varvec{x}}_i,1,Y_i^{obs}(1),Y_i(0)): i \in \mathcal {I}_t\}$, where the responses under the treatment are observed; and (iii) $\{({\varvec{x}}_i,T_i,Y_i(1),Y_i(0)): i \in \mathcal {I}_m\}$, where only covariates are observed. Here, we use $\mathcal {I}_c$, $\mathcal {I}_t$, and $\mathcal {I}_m$ to denote the index sets of the subjects in the respective categories and, therefore, $\mathcal {I}_c\cup \mathcal {I}_t\cup \mathcal {I}_m=\{1,\dots ,n_{test}\}$. For the ITE of each subject in the test set, we can construct the prediction interval with a desired confidence level of $1-\alpha$ in the following procedure:

(i) *For subject *$i\in \mathcal {I}_c$: At each iteration *k* of Algorithm 1, calculate the prediction $\hat{Y}_i^{(k)}(1)=\hat{c}^{(k)}({\varvec{x}}_i)+ \hat{\tau }^{(k)}({\varvec{x}}_i)+\hat{\sigma }^{(k)} Z_{new}^{(k,1)}$, where $Z_{new}^{(k,1)}\sim N(0,1)$. Let $c_l({\varvec{x}}_i,1)$ and $c_u({\varvec{x}}_i,1)$ denote, respectively, the $\frac{\alpha }{2}$- and $(1-\frac{\alpha }{2})$-quantiles of $\{\hat{Y}_i^{(k)}(1): k=\mathcal {K}+1, \mathcal {K}+2,\ldots , \mathcal {K}+M\}$ collected over iterations. Since $Y_i^{obs}(0)$ is observed, $(c_l({\varvec{x}}_i,1)-Y_i^{obs}(0),c_u({\varvec{x}}_i,1)-Y_i^{obs}(0))$ forms a $(1-\alpha )$-prediction interval for the ITE $Y_i(1)-Y_i^{obs}(0)$.
(ii) *For subject *$i\in \mathcal {I}_t$: At each iteration *k* of Algorithm 1, calculate the prediction $\hat{Y}_i^{(k)}(0)=\hat{c}^{(k)}({\varvec{x}}_i)+\hat{\sigma }^{(k)} Z_{new}^{(k,2)}$, where $Z_{new}^{(k,2)}\sim N(0,1)$. Let $c_l({\varvec{x}}_i,0)$ and $c_u({\varvec{x}}_i,0)$ denote, respectively, the $\frac{\alpha }{2}$- and $(1-\frac{\alpha }{2})$-quantiles of $\{\hat{Y}_i^{(k)}(0): k=\mathcal {K}+1, \mathcal {K}+2,\ldots , \mathcal {K}+M\}$ collected over iterations. Since $Y_i^{obs}(1)$ is observed, $(Y_i^{obs}(1)-c_u({\varvec{x}}_i,0), Y_i^{obs}(1)-c_l({\varvec{x}}_i,1))$ forms a $(1-\alpha )$-prediction interval for the ITE $Y_i^{obs}(1)-Y_i(0)$.
(iii) *For subject *$i\in \mathcal {I}_m$: At each iteration *k* of Algorithm 1, calculate the prediction $\hat{Y}_i^{(k)}(1)-\hat{Y}_i^{(k)}(0)=\hat{\tau }^{(k)}({\varvec{x}}_i)+\sqrt{2} \hat{\sigma }^{(k)} Z_{new}^{(k,3)}$, where $Z_{new}^{(k,3)}\sim N(0,1)$. Let $c_l({\varvec{x}}_i)$ and $c_u({\varvec{x}}_i)$ denote, respectively, the $\frac{\alpha }{2}$- and $(1-\frac{\alpha }{2})$-quantiles of $\{ \hat{Y}_i^{(k)}(1)-\hat{Y}_i^{(k)}(0): k=\mathcal {K}+1, \mathcal {K}+2,\ldots , \mathcal {K}+M\}$ collected over iterations. Then $(c_l({\varvec{x}}_i), c_u({\varvec{x}}_i))$ forms a $(1-\alpha )$-prediction interval for the ITE $Y_i(1)-Y_i(0)$.

### Simulation Study

#### Example 1

Consider the data-generating equation

24 $$\begin{aligned} y_i=\mu +{\varvec{x}}_i^{\top } {\varvec{\beta }}+(\eta _0 +\eta ({\varvec{x}}_i)) T_i +\sigma z_i, \quad i=1,2,\ldots ,n, \end{aligned}$$

where ${\varvec{x}}_i=(x_{i,1},x_{i,2})^{\top }$ with each element drawn independently from *Unif*(0, 1), $\mu =1$, ${\varvec{\beta }}=(1,1)^{\top }$, $\eta _0=1$, $\sigma =1$, $z_i \sim N(0,1)$, and $\eta ({\varvec{x}}_i)=s(x_{i1})s(x_{i2})-E(s(x_{i1})s(x_{i2}))$. As in Lei and Candès (2021), we set $s(a)=\frac{2}{1+exp(-12(a-0.5))}$, and generate the treatment variable $T_i$ according to the propensity score model:

25 $$\begin{aligned} e({\varvec{x}}_i)=\frac{1}{4}(1+\beta _{2,4}(x_{i,1})), \end{aligned}$$

where $\beta _{2,4}$ is the CDF of the beta distribution with parameters (2,4), ensuring $e({\varvec{x}}_i) \in [0.25,0.5]$ and thereby sufficient overlap between the treatment and control groups. In terms of equation (1), we have $c({\varvec{x}}_i)=\mu +{\varvec{x}}_i^{\top }{\varvec{\beta }}$ and $\tau ({\varvec{x}}_i)=\eta _0 +\eta ({\varvec{x}}_i)$. We generated 20 datasets from the model (24) independently, each consisting of $n_{train}=500$ training samples and $n_{test}=1000$ test samples.

**Table 2:** Comparison of Double-NN and CQR for inference of the predictive ITE for Example (24), where the coverage and length of the prediction intervals were calculated by averaging over 20 datasets with the standard deviation given in the parentheses

	Case $\mathcal {I}_c$		Case $\mathcal {I}_t$			Case $\mathcal {I}_m$	
Method	Coverage	Length	Coverage	Length	Method	Coverage	Length
Double-NN	0.9549	4.2004	0.9581	4.1812	Double-NN	0.9583	5.6056
	(0.0095)	(0.1567)	(0.0098)	(0.1541)		(0.0103)	(0.2207)
CQR-BART	0.9472	4.2702	0.9533	4.4024	CQR(inexact)	0.9530	6.3244
	(0.0342)	(0.5225)	(0.0341)	(0.8972)		(0.0198)	(0.5426)
CQR-Boosting	0.9556	5.5199	0.9548	4.4493	CQR(exact)	1.0000	13.4005
	(0.0294)	(0.5866)	(0.0259)	(0.5097)		(0.0002)	(2.4936)
CQR-RF	0.9529	5.4609	0.9652	4.6428	CQR(naive)	0.9998	12.8861
	(0.0233)	(0.5172)	(0.0171)	(0.5408)		(0.0004)	(1.5275)
CQR-NN	0.9570	6.4072	0.9755	5.8125			
	(0.0195)	(0.8087)	(0.0199)	(1.4332)			

For this example, we assume the functional form of $c({\varvec{x}})$ is known and model $\tau ({\varvec{x}})$ by a DNN. The DNN has two hidden layers, each consisting of 10 hidden neurons. The number of parameters of the DNN is $|{\varvec{\theta }}_{\tau }|=151$, and the total dimension of ${\varvec{\theta }}=(\mu , \eta _0,{\varvec{\beta }},{\varvec{\theta }}_{\tau }^{\top },\log (\sigma ))^{\top }$ is 156 $(\approx n_{train}^{0.81})$, which falls into the class of large models.

Refer to Section §3 of the supplement for parameter settings for the Double-NN method. For comparison, the conformal quantile regression (CQR) method (Romano et al. 2019; Lei and Candès 2021) was applied to this example, where the outcome function was approximated using different machine learning methods, including BART (Chipman et al. 2010), Boosting (Schapire 1990; Breiman 1998), and random forest (RF) (Breiman 2001), and neural network (NN). Refer to Section §2 of the supplement for a brief description of the CQR method. For CQR-NN, we used a neural network of structure $(p+1)$-10-10-2 to model the outcome quantiles, where the extra input variable is for treatment and the two output neurons are for $(\alpha /2,1-\alpha /2)$-quantiles of the outcome (Romano et al. 2019). Additionally, we used a neural network of structure *p*-10-10-1 to model the propensity score in order to compute weighted CQR as in Lei and Candès (2021).

The other CQR methods were implemented using the R package *cfcausal* (Lei and Candès 2021). For the case $\mathcal {I}_m$, we considered CQR-BART only, given its relative superiority over other CQR methods in the cases $\mathcal {I}_c$ and $\mathcal {I}_t$.

The results were summarized in Table 2. The comparison shows that the Double-NN method outperforms the CQR methods in both the coverage rate and length of the prediction intervals under all the three cases $\mathcal {I}_c$, $\mathcal {I}_t$, and $\mathcal {I}_m$. Specifically, the prediction intervals resulting from the Double-NN method tend to be shorter, while their coverage rates tend to be closer to the nominal level.

**Fig. 2:**
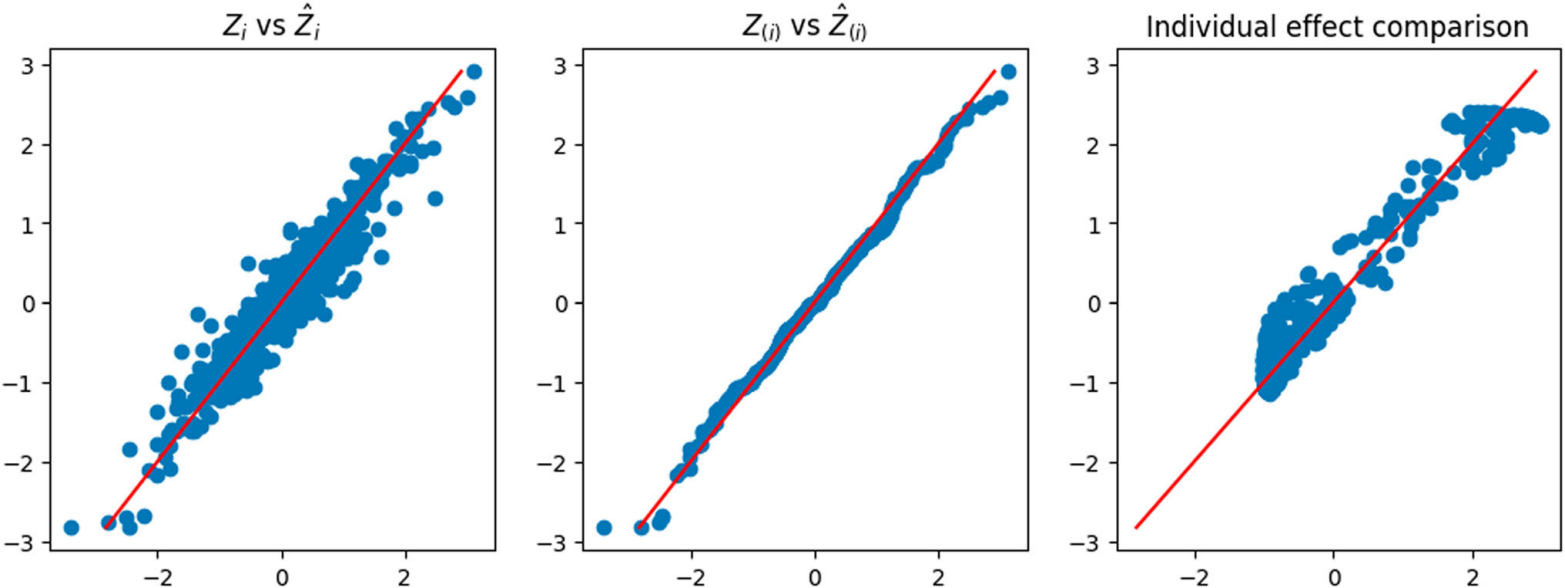
Demonstration of the Double-NN method for a dataset simulated from (24): (left) scatter plot of $\hat{{\varvec{z}}}_i$ (*y*-axis) versus ${\varvec{z}}_i$ (*x*-axis); (middle) Q-Q plot of $\hat{{\varvec{z}}}_i$ and ${\varvec{z}}_i$; (right) scatter plot of $\tau ({\varvec{x}}_i)$ (*y*-axis) versus $\hat{\tau }({\varvec{x}}_i)$ (*x*-axis)

Figure 2 demonstrates the rationale underlying the Double-NN method. The left scatter plot compares the imputed and true values of the latent variables for a dataset simulated from (24), where the imputed values were collected at the last iteration of Algorithm 1. The comparison reveals a close match between the imputed and true latent variable values, with the variability of the imputed values representing the source of uncertainty in the data-generating system. This variability in the latent variables can be propagated to ${\varvec{\theta }}$ through the estimated inverse function $G(\cdot )$, leading to the uncertainty in parameters and, consequently, the uncertainty in predictions. The middle scatter plot shows that the imputed latent variable values follows the standard Gaussian distribution, as expected. The right scatter plot compares the estimated and true values of the function $\tau ({\varvec{x}}_i)$, with the variability of the estimator representing its uncertainty. This plot further implies that the Double-NN method not only works for performing inference for the predictive ITE but also works for performing inference for CATE.

#### Example 2

Consider the data-generating equation

26 $$\begin{aligned} y_i=c({\varvec{x}}_i)+\tau ({\varvec{x}}_i) T_i +\sigma z_i, \quad i=1,2,\ldots ,n, \end{aligned}$$

where ${\varvec{x}}_i=(x_{i,1},x_{i,2},\ldots ,x_{i,5})^{\top }$ with each element drawn independently from *Unif*(0, 1), $\tau ({\varvec{x}})$ and $T_i$ are generated as in Example 1 except that ${\varvec{x}}_i$ contains three extra false covariates, $c({\varvec{x}}_i)=\frac{2x_{i,1}}{1+5x_{i,2}^2}$, $\sigma =1$, and $z_i \sim N(0,1)$. We simulated 20 datasets from this equation, each consisting of $n_{train}=1000$ training samples and $n_{test}=1000$ test samples.

For this example, we modeled both $c({\varvec{x}})$ and $\tau ({\varvec{x}})$ using DNNs. Each of the DNNs consists of two hidden layers, each layer consisting of 10 hidden neurons. In consequence, ${\varvec{\theta }}=({\varvec{\theta }}_c^{\top },{\varvec{\theta }}_{\tau }^{\top },\log (\sigma ))^{\top }$ has a total dimension of 363 $(\approx n_{train}^{0.85})$.

Similar to Example 1, we also applied the CQR methods (Lei and Candès 2021) to this example for comparison. The CQR methods were implemented as described in Example 1. The results were summarized in Table 3, which indicates again that the Double-NN method outperforms the CQR methods under all the three cases $\mathcal {I}_c$, $\mathcal {I}_t$, and $\mathcal {I}_m$. The prediction intervals resulting from the Double-NN method tend to be shorter, while their coverage rates tend to be closer to the nominal level.

**Fig. 3:**
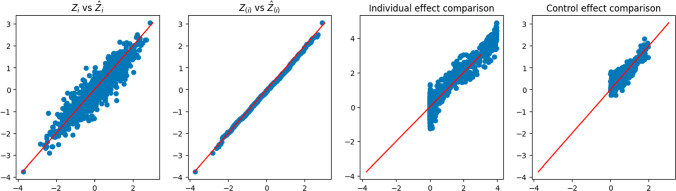
Demonstration of the Double-NN method for a dataset simulated from (26): (left) scatter plot of $\hat{{\varvec{z}}}_i$ (*y*-axis) versus ${\varvec{z}}_i$ (*x*-axis); (middle-left) Q-Q plot of $\hat{{\varvec{z}}}_i$ and ${\varvec{z}}_i$; (middle-right) scatter plot of $c({\varvec{x}}_i)$ (*y*-axis) versus $\hat{c}({\varvec{x}}_i)$ (*x*-axis); (right) scatter plot of $\tau ({\varvec{x}}_i)$ (*y*-axis) versus $\hat{\tau }({\varvec{x}}_i)$ (*x*-axis)

Similar to Figure 2, Figure 3 demonstrates the rationale underlying the Double-NN method, as well as its capability for CATE inference. The left plot demonstrates the variability embedded in the latent variables of the data-generating system. The middle-left plot shows that the imputed latent variables are distributed according to the standard Gaussian distribution, as expected. The right two plots display the estimates of $c({\varvec{x}}_i)$ and $\tau ({\varvec{x}}_i)$, respectively. Once again, we note that the variations of the estimates of $c({\varvec{x}}_i)$ and $\tau ({\varvec{x}}_i)$, as depicted in their respective scatter plots, reflect their uncertainty according to the theory of EFI.

**Table 3:** Comparison of Double-NN and CQR for inference of the predictive ITE for Example (26), where the coverage and length of the prediction intervals were calculated by averaging over 20 datasets with the standard deviation given in the parentheses.

	Case $\mathcal {I}_c$		Case $\mathcal {I}_t$			Case $\mathcal {I}_m$	
Method	Coverage	Length	Coverage	Length	Method	Coverage	Length
Double-NN	0.9519	4.2727	0.9645	4.246	Double-NN	0.9604	6.0079
	(0.0111)	(0.0101)	(0.0069)	(0.0967)		(0.0946)	(0.1363)
CQR-BART	0.9584	4.3586	0.9545	4.2658	CQR(inexact)	0.9386	6.0492
	(0.0220)	(0.4392)	(0.0230)	(0.4586)		(0.0270)	(0.6062)
CQR-Boosting	0.9536	4.9942	0.9572	4.4393	CQR(exact)	0.9996	12.1252
	(0.0175)	(0.4044)	(0.0194)	(0.4213)		(0.0007)	(1.1022)
CQR-RF	0.9563	5.6658	0.9580	4.4399	CQR(naive)	0.9988	11.5566
	(0.0198)	(0.4777)	(0.0232)	(0.5044)		(0.0014)	(0.9309)
CQR-NN	0.9595	4.6748	0.9452	3.9579			
	(0.0165)	(0.6015)	(0.0185)	(0.4301)			

Precision in Estimation of Heterogeneous Effects As demonstrated in Figure 2 and Figure 3, the Double-NN method can also be used for inference of CATE. The performance in CATE estimation is often measured using the expected Precision in Estimation of Heterogeneous Effects (PEHE), which is defined as:

$$\begin{aligned} \epsilon _{PEHE}=\int _{\mathcal {X}}(\hat{\tau }({\varvec{x}})-\tau ({\varvec{x}}))^2 dF({\varvec{x}}), \end{aligned}$$

where $F({\varvec{x}})$ denotes the distribution function of the covariates ${\varvec{X}}$. As we can see, $\epsilon _{PEHE}$ summarizes the precision of the CATE over the entire sample space $\mathcal {X}$ (Hill 2011; Shalit et al. 2017; Caron et al. 2022). In practice, since we only observe the treatment effect on the treatment group, the target of interest is generally only for the treatment group, i.e $\epsilon _{PEHE}^{(T)}=\int _{\mathcal {X}}(\hat{\tau }({\varvec{x}})- \tau ({\varvec{x}}))^2 dF_T({\varvec{x}})$, where $F_T({\varvec{x}})$ denotes the distribution function of the covariates in the treatment group. We estimated $\epsilon _{PEHE}^{(T)}$ by $\hat{\epsilon }_{PEHE}^{(T)}=\frac{1}{n_t}\sum _{i\in I_{t}}(\hat{\tau }({\varvec{x}}_i)-\tau ({\varvec{x}}_i))^2$. For the Double-NN method, we set $\hat{\tau }({\varvec{x}}_i)=\frac{1}{M}\sum _{k=\mathcal {K}+1}^{\mathcal {K}+M}\hat{\tau }^{(k)}({\varvec{x}}_i)$, where *M* denotes the number of estimates of $\tau ({\varvec{x}}_i)$ collected in a run of Algorithm 1.

For comparison, the existing CATE estimation methods, including single-learner (S-learner), two-learner (T-learner), and X-learner (Künzel et al. 2019), have been applied to the datasets generated above, where the RF and BART are used as the base learners. In the S-learner, a single outcome function is estimated using a base learner with all available covariates, where the treatment indicator is treated as a covariate, and then estimate CATE by $\hat{\tau }_S=\hat{\mu }({\varvec{x}},1)-\hat{\mu }({\varvec{x}},0)$, where $\hat{\mu }({\varvec{x}},t)$ denotes the outcome function estimator. The T-learner estimates the outcome functions using a base learner separately for the units under the control and those under the treatment, and then estimate CATE by $\hat{\tau }_T({\varvec{x}})=\hat{\mu }_1({\varvec{x}})-\hat{\mu }_0({\varvec{x}})$, where $\hat{\mu }_t({\varvec{x}})$ denote the outcome function estimator for the assignment group $t \in \{0,1\}$. The X-learner builds on the T-learner; it uses the observed outcomes to estimate the unobserved ITEs, and then estimate the CATE in another step as if the ITEs were observed. Refer to Künzel et al. (2019) and Caron et al. (2022) for the detail. We implemented the S-learner, T-learner, and X-leaner using the package downloaded at https://github.com/albicaron/EstITE.

Table 4 compares the values of $\hat{\epsilon }_{PEHE}^{(T)}$ resulting from the Double-NN, S-learners, T-learners, and X-learners. for the models (24) and (26). The comparison shows that the Double-NN method outperforms the existing ones in achieving consistent CATE estimates over different covariate values. This is remarkable! As explained in Section 4, we would attribute this performance of the Double-NN method to its fidelity in parameter estimation (Liang et al. 2024). Compared to the MLE method, which serves as the prototype for the base learners, the Double-NN method is forced to be more robust to covariates due to added penalty term $U_n({\varvec{Y}}_n,{\varvec{X}}_n,{\varvec{Z}}_n, {\varvec{w}}_n)/\epsilon$.

**Table 4:** Comparison of Double-NN and other methods in $\epsilon _{PEHE}^{(T)}$, where each of the mean and standard deviations was calculated based on 20 datasets generated from (24) or (26)

	Model (24)		Model (26)	
Method	Training	Test	Training	Test
S-RF	0.3769 ± 0.0170	0.3660 ± 0.0188	0.3722 ± 0.0074	0.3377 ± 0.0100
S-BART	0.4233 ± 0.0156	0.4344 ± 0.0149	0.3371 ± 0.0099	0.3418 ± 0.0102
T-RF	0.4545 ± 0.0114	0.4198 ± 0.0118	0.4095 ± 0.0064	0.3488 ± 0.0084
T-BART	0.4190 ± 0.0139	0.4236 ± 0.0127	0.4308 ± 0.0092	0.4298 ± 0.0093
X-RF	0.3416 ± 0.0153	0.3451 ± 0.0162	0.2761 ± 0.0106	0.2789 ± 0.0106
X-BART	0.3863 ± 0.0137	0.3972 ± 0.0128	0.3853 ± 0.0102	0.3862 ± 0.0097
Double-NN	0.2962 ± 0.0167	0.3139 ± 0.0178	0.3788 ± 0.0105	0.3899 ± 0.0110

### Real Data Analysis

#### Lalonde

The ‘LaLonde’ data is a well-known dataset used in causal inference to evaluate the effectiveness of a job training program in improving the employment prospects of participants. We used the dataset given in the package “twang” (Cefalu et al. 2021) among various versions. The dataset includes earning data in 1978 on 614 individuals, with 185 receiving job training and 429 in the control group. There are 8 covariates including various demographic, educational, and employment-related variables. While the LaLonde dataset has been widely used for ATE estimation, we use it to illustrate the Double-NN method for constructing ITE prediction intervals.

To evaluate the performance of different methods, we randomly split the LaLonde dataset into a training set and a test set. The training set, denoted by $\mathcal {D}_{train}$, consists of $n_{train}=600$ observations; while the test set, denoted by $\mathcal {D}_{test}$, consists of $n_{test}=14$ observations. We trained the Double-NN on $\mathcal {D}_{train}$ and constructed prediction intervals for each subject in $\mathcal {D}_{test}$ with a confidence level of $1-\alpha =0.5$. For the Double-NN, we modeled both $c({\varvec{x}})$ and $\tau ({\varvec{x}})$ using DNNs. Each of the DNNs consists of two hidden layers, with each layer consisting of 10 hidden neurons. In consequence, ${\varvec{\theta }}=({\varvec{\theta }}_c^{\top },{\varvec{\theta }}_{\tau }^{\top },\log (\sigma ))^{\top }$ has a dimension of 423 $(\approx n_{train}^{0.95})$, a challenging task for uncertainty quantification of the model.

**Fig. 4:**
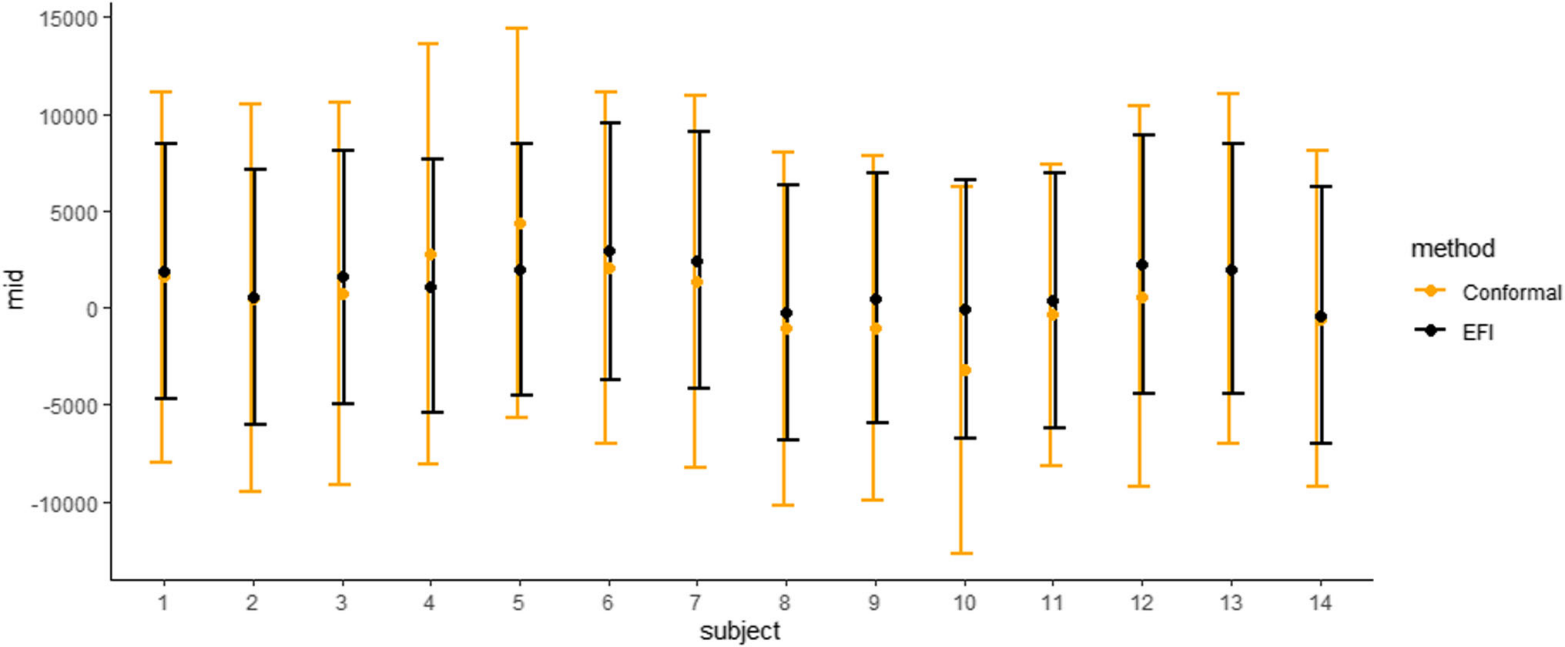
Comparison of prediction intervals resulting from Double-NN (labeled as EFI) and CQR (labeled as conformal) for the subjects in the test set of Lalonde

Figure 4 displays the constructed ITE prediction intervals for the test data, comparing the proposed method to the CQR method (Lei and Candès 2021). The comparison shows that the prediction intervals resulting from the proposed method are shorter than those from the CQR method, while the centers of those intervals are similar. This suggests that the proposed method is able to estimate the ITEs with a higher degree of precision.

#### NLSM

This subsection conducts an analysis on the ‘National Study of Learning Mindsets’ (NLSM) dataset used in the 2018 Atlantic Causal Inference Conference workshop (Yeager et al. 2019; Carvalho et al. 2019). NSLM records the results of a randomized evaluation for a “nudge-like” intervention designed to instill students with a growth mindset. The dataset is available at https://github.com/grf-labs/grf/tree/master/experiments/acic18, which includes 10,391 students from 76 schools, with four student-level covariates and six school-level students. After factoring the categorical variables, the dimension of covariates ${\varvec{x}}$ increases to 29.

Due to unavailability of the true treatment effect values, we performed an exploratory analysis as in Lei and Candès (2021). In order to construct prediction intervals for the ITE, we split the dataset into two sets: $\mathcal {D}_{train}$ and $\mathcal {D}_{test}$. The former has a sample size of $n_{train}=5200$, and the latter has a sample size of $n_{test}=5191$. For the Double-DNN method, we used DNNs to model the functions $\tau ({\varvec{x}})$ and $c({\varvec{x}})$. Each DNN consists of two hidden layers, with each hidden layer consisting of 10 hidden neurons. Therefore, the dimension of ${\varvec{\theta }}=({\varvec{\theta }}_c^{\top },{\varvec{\theta }}_{\tau }^{\top },\log (\sigma ))^{\top }$ is 843 $(\approx n_{train}^{0.79})$.

The Double-DNN was trained on $\mathcal {D}_{train}$ and the prediction intervals were constructed on $\mathcal {D}_{test}$, which corresponds to case (iii) described in Section 5.1. This process was repeated 20 times. For comparison, the CQR method (Lei and Candès 2021) was also applied to this example.

**Fig. 5:**
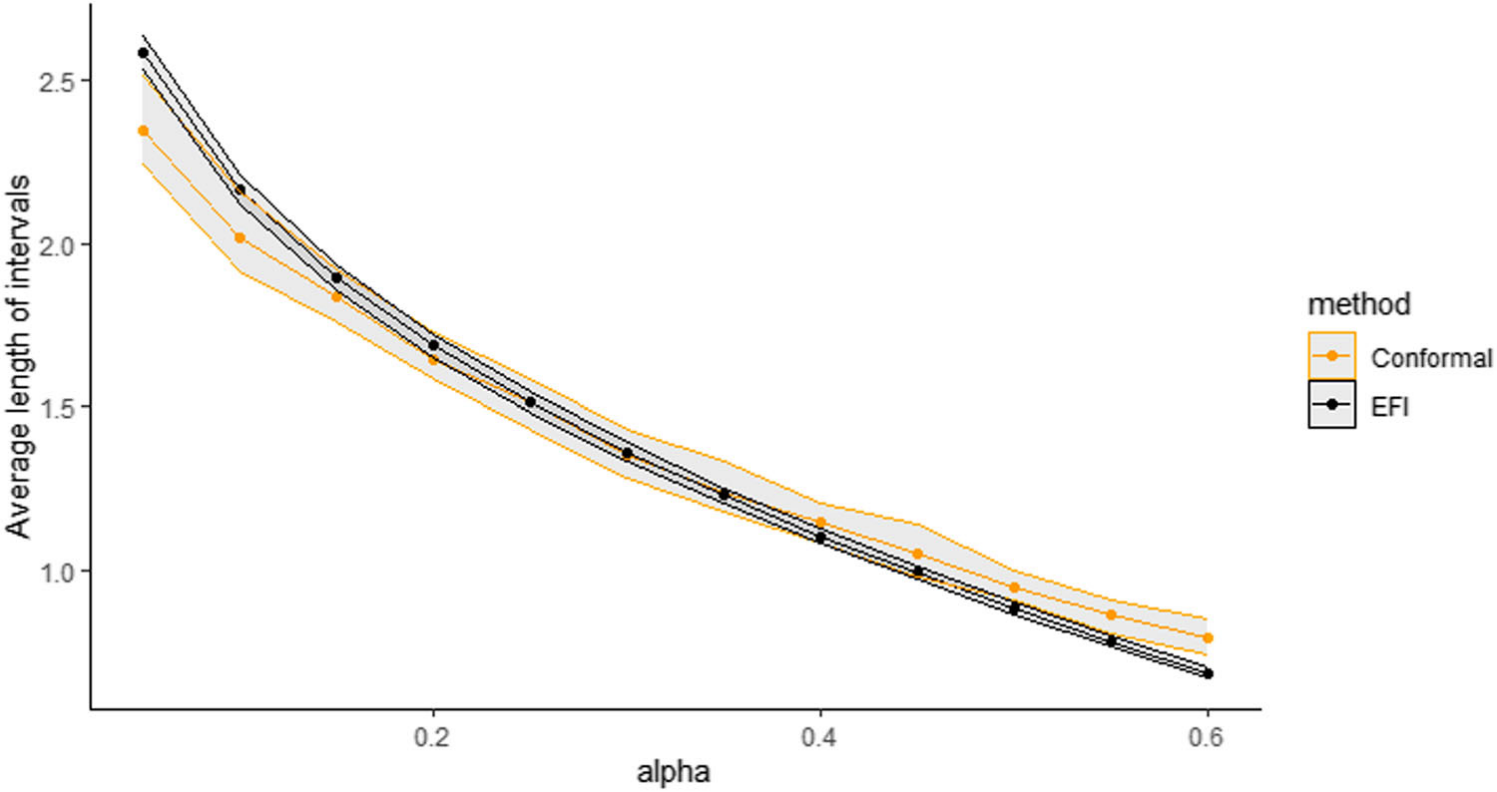
Comparison of the average length of intervals obtained by the Double-NN (labeled as EFI) and CQR (labeled as conformal) for the NLSM data

**Fig. 6:**
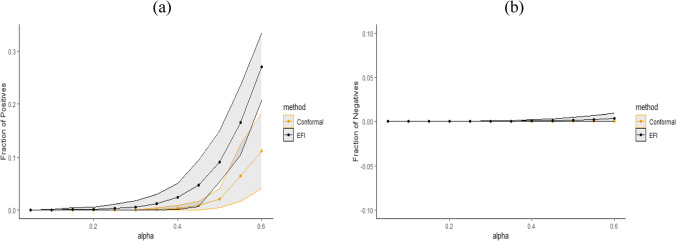
Fractions of the intervals obtained by Double-NN (labeled as EFI) and CQR (labeled as conformal) with (a) positive lower bounds and (b) negative upper bounds, where the upper and lower envelops are respectively $95\%$ and $5\%$ quantiles across 20 runs

Figure 5 displays the average length of prediction intervals, obtained by Double-DNN and CQR, as a function of $\alpha$, with the upper and lower envelops being respectively the $95\%$ and $5\%$ quantiles across 20 runs. For this example, we implemented CQR using the “inexact” method, and therefore, its interval lengths tend to be short with approximate validity. However, as shown in Figure 5, the prediction intervals resulting from the Double-NN method tend to be even shorter than those from CQR as $\alpha$ increases. Figure 6 (a) compares the fractions of the prediction intervals, obtained by Double-NN and CQR, that cover positive values only. While Figure 6 (b) compares the fractions of the prediction intervals that cover negative values only. In summary, the Double-NN can provide more accurate predictions for the ITE than CQR for this example. Specifically, the Double-NN identified fewer subjects with significant ITEs than the CQR, as implied by Figure 6 (a) and (b); while each has a narrow prediction interval, as implied by Figure 5.

## Discussion

This paper extends EFI to statistical inference for large statistical models and applies the proposed Double-NN method to treatment effect estimation. The numerical results demonstrate that the Double-NN method significantly outperforms the existing CQR method in ITE prediction. As mentioned in the paper, we attribute the superior performance of the Double-NN method to its fidelity in parameter estimation. Due to the universal approximation ability of deep neural networks, the Double-NN method is generally applicable for causal effect estimation.

From the perspective of statistical inference, this paper advances the theory and methodology for making inference of large statistical models, allowing the model size to increase with the sample size *n* at a rate of $O(n^{\zeta })$ for any exponent $0 \le \zeta <1$. In particular, the Double-NN method provides a rigorous approach for quantifying the uncertainty of deep neural networks. In this paper, we have tested the performance of the Double-NN method on numerical examples with the exponent ranging $0.79 \le \zeta \le 0.95$, which all falls into the class of large models.

The Double-NN method can be further extended toward a general nonparametric approach for causal inference. Specifically, we can include an additional neural network to approximate the propensity score, enabling the outcome and propensity score functions to be simultaneously estimated. This extension will enable the use of inverse probability weighting methods to further improve ATE estimation, especially in the scenario where the covariate distributions in the treatment and control groups are imbalanced (Shalit et al. 2017; Hahn et al. 2020). From the perspective of EFI, this just corresponds to making inference for a different $b({\varvec{\theta }})$ function. Similarly, for inference of ITE, a different $b({\varvec{\theta }})$ function, including those adjusted with propensity scores, can also be used. The key advantage of EFI is its ability to automatically quantify the uncertainty of these functions as prescribed in (20), even when the functions are highly complex.

Regarding the size of large models, our theory does not preclude applications to large-scale DNNs with millions or even billions of parameters, as supported by the neural scaling law. As mentioned previously, Hestness et al. (2017) investigated the relationship between the DNN model size and the dataset size: they discovered a sub-linear scaling law of $dim({\varvec{\theta }}) \prec n$ across various model architectures in machine learning applications, including machine translation, language modeling, image processing, and speech recognition. Their findings suggest that Theorem 3.1 remains valid for large-scale DNNs by choosing an appropriate growth rate for their depth.

In practice, we often encounter small-*n*-large-*p* problems. For such a problem, we need to deal with a model of dimension $dim({\varvec{\theta }}) \succeq n$, which is often termed as an over-parameterized model. A further extension of EFI for over-parameterized models is possible by imposing an appropriate sparsity constraint on ${\varvec{\theta }}$. How to make post-selection inference with EFI for the over-parameterized models will be studied in future work.

Finally, we note that a recent work by Williams (2023) demonstrates how conformal prediction sets arise from a generalized fiducial distribution. Given the inherent connections between GFI and EFI, we believe that the results established in Williams (2023) should also apply to EFI. In particular, EFI follows the same switching principle as GFI (Hannig et al. 2016), which infers the uncertainty of the model parameters from the distribution of unobserved random errors. Further research on EFI from this perspective is of great interest, as it could potentially alleviate EFI’s reliance on assumptions about the underlying data distribution in prediction uncertainty quantification.

## Supplementary Material

This material provides (i) the proof for Theorem 3.1, (ii) a brief description for the CQR method, and (iii) the parameter settings for the experiments reported in the paper.

## Supplementary Information

Below is the link to the electronic supplementary material.

Supplementary file 1 (pdf 402 KB)

## Data Availability

Data is provided within the manuscript file.
