# Supplementary material for: Extended fiducial inference for individual treatment effects via deep neural networks
Source: Stat Comput. 2025 May 17;35(4):97. doi: 10.1007/s11222-025-10624-8 (PMC12085359; doi:10.1007/s11222-025-10624-8)
Supplement: Supplementary file 1 — (pdf 402 KB) [file 11222_2025_10624_MOESM1_ESM.pdf]

Supplement for "Extended Fiducial Inference for Individual Treatment Effects via Deep Neural Networks"

Sehwan Kim† and Faming Liang*

April 18, 2025

This material is organized as follows. Section §1 provides a proof for Theorem 3.1. Section §2 provides a brief description for the CQR method. Section §3 provides parameter settings for the experiments reported in the main text and this supplement.

§1 Theoretical Proofs

To prove the validity of the proposed method, it is sufficient to prove that $\hat{g}(\cdot)$ constitutes a consistent estimator of $\boldsymbol{\theta}$, building on the theory developed in Liang et al. (2024). To ensure the self-contained nature of this paper, we provide a concise overview of the theory presented in Liang et al. (2024) in Section §1.1 of this supplement. Subsequently, our study will center on establishing the consistency of $\hat{g}(\cdot)$.

§1.1 Outline of the Proof

First of all, we note that the theoretical study is conducted under the assumption that the EFI network has been correctly specified such that a sparse EFI network $\tilde{\boldsymbol{w}}_n^*$ exists, from which the complete data $(\boldsymbol{X}_n, \boldsymbol{Y}_n, \boldsymbol{Z}_n^*)$ can be generated, where $\boldsymbol{Z}_n^*$ represents the values of the latent variables realized in the observed samples. Specifically, we assume $\boldsymbol{Z}_n^* \sim \pi_\epsilon(\boldsymbol{Z}|\boldsymbol{X}_n, \boldsymbol{Y}_n, \tilde{\boldsymbol{w}}_n^*)$ holds as $\epsilon \downarrow 0$.

For the EFI network, we define

$$\widehat{\mathcal{G}}(\boldsymbol{w}_n|\tilde{\boldsymbol{w}}_n^*) := \frac{1}{n}\log \pi_\epsilon(\boldsymbol{Y}_n, \boldsymbol{Z}_n^*|\boldsymbol{X}_n, \boldsymbol{w}_n) + \frac{1}{n}\log \pi(\boldsymbol{w}_n). \tag{S1}$$

Therefore,

$$\hat{\boldsymbol{w}}_n^* := \arg\max_{\boldsymbol{w}_n \in \mathcal{W}_n} \widehat{\mathcal{G}}(\boldsymbol{w}_n|\tilde{\boldsymbol{w}}_n^*),$$

*Correspondence author: Faming Liang, email: fmliang@purdue.edu. † Department of Statistics, Ewha Womans University, Seoul 03760, Republic of Korea. * Department of Statistics, Purdue University, West Lafayette, IN 47907, USA.

is the global maximizer of the posterior $\pi_\epsilon(\boldsymbol{w}_n|\boldsymbol{X}_n, \boldsymbol{Y}_n, \boldsymbol{Z}_n^*)$. Also, we define

$$\begin{aligned}\widetilde{\mathcal{G}}(\boldsymbol{w}_n|\tilde{\boldsymbol{w}}_n^*) &:= \frac{1}{n}\int \log \pi_\epsilon(\boldsymbol{Y}_n, \boldsymbol{Z}_n^*|\boldsymbol{X}_n, \boldsymbol{w}_n) d\pi_\epsilon(\boldsymbol{Z}_n^*|\boldsymbol{X}_n, \boldsymbol{Y}_n, \tilde{\boldsymbol{w}}_n^*) + \frac{1}{n}\log \pi(\boldsymbol{w}_n). \\ &= \frac{1}{n}\Big\{ \log \pi(\boldsymbol{w}_n|\boldsymbol{X}_n, \boldsymbol{Y}_n) - \int \log \frac{\pi(\boldsymbol{Z}_n^*|\boldsymbol{X}_n, \boldsymbol{Y}_n, \tilde{\boldsymbol{w}}_n^*)}{\pi(\boldsymbol{Z}_n^*|\boldsymbol{X}_n, \boldsymbol{Y}_n, \boldsymbol{w}_n)} d\pi(\boldsymbol{Z}_n^*|\boldsymbol{X}_n, \boldsymbol{Y}_n, \tilde{\boldsymbol{w}}_n^*) \\ &+ \int \log \pi(\boldsymbol{Z}_n^*|\boldsymbol{X}_n, \boldsymbol{Y}_n, \tilde{\boldsymbol{w}}_n^*) d\pi(\boldsymbol{Z}_n^*|\boldsymbol{X}_n, \boldsymbol{Y}_n, \tilde{\boldsymbol{w}}_n^*) + c\Big\},\end{aligned} \tag{S2}$$

where $c = \log \int_{\mathcal{W}_n} \pi(\boldsymbol{Y}_n|\boldsymbol{X}_n, \boldsymbol{w}_n)\pi(\boldsymbol{w}_n)d\boldsymbol{w}_n$ is the log-normalizing constant of the posterior $\pi(\boldsymbol{w}_n|\boldsymbol{X}_n, \boldsymbol{Y}_n)$. Note that in the above derivation, $\boldsymbol{X}_n$ can be ignored for simplicity since it is constant. For simplicity of notation, we let

$$D_{KL}(\boldsymbol{w}_n) = \int \log \frac{\pi(\boldsymbol{Z}_n^*|\boldsymbol{X}_n, \boldsymbol{Y}_n, \tilde{\boldsymbol{w}}_n^*)}{\pi(\boldsymbol{Z}_n^*|\boldsymbol{X}_n, \boldsymbol{Y}_n, \boldsymbol{w}_n)} d\pi(\boldsymbol{Z}_n^*|\boldsymbol{X}_n, \boldsymbol{Y}_n, \tilde{\boldsymbol{w}}_n^*),$$

be the Kullback-Leibler divergence between $\pi(\boldsymbol{Z}_n^*|\boldsymbol{X}_n, \boldsymbol{Y}_n, \tilde{\boldsymbol{w}}_n^*)$ and $\pi(\boldsymbol{Z}_n^*|\boldsymbol{X}_n, \boldsymbol{Y}_n, \boldsymbol{w}_n)$. Regarding the EFI network, we make the following assumption:

Assumption 1 *The EFI network satisfies the conditions:*

(i) The parameter space $\mathcal{W}_n$ (of $\boldsymbol{w}_n$) is convex and compact.

(ii) $\mathbb{E}(\log \pi_\epsilon(Y, Z|X, \boldsymbol{w}_n))^2 < \infty$ for any $\boldsymbol{w}_n \in \mathcal{W}_n$, where (X, Y, Z) denotes a generic sample as those in $(\boldsymbol{Y}_n, \boldsymbol{Z}_n, \boldsymbol{X}_n)$.

Define $Q^*(\boldsymbol{w}_n) = \mathbb{E}(\log \pi_\epsilon(Y, Z|X, \boldsymbol{w}_n)) + \frac{1}{n}\log \pi(\boldsymbol{w}_n)$. By Assumption 1 and the weak law of large numbers,

$$\frac{1}{n}\log \pi_\epsilon(\boldsymbol{w}_n|\boldsymbol{X}_n, \boldsymbol{Y}_n, \boldsymbol{Z}_n) - Q^*(\boldsymbol{w}_n) \xrightarrow{p} 0, \tag{S3}$$

holds uniformly over the parameter space $\mathcal{W}_n$, where $\xrightarrow{p}$ denotes convergence in probability. Further, we make the following assumption on $Q^*(\boldsymbol{w}_n)$, which is essentially an identifiability condition of $\hat{\boldsymbol{w}}_n^*$.

Assumption 2 *(i) $Q^*(\boldsymbol{w}_n)$ is continuous in $\boldsymbol{w}_n$ and is uniquely maximized at some point $\boldsymbol{w}_n^\diamond$; (ii) for any $\epsilon > 0$, the value $sup_{\boldsymbol{w}_n \in \mathcal{W}_n \setminus B(\epsilon)} Q^*(\boldsymbol{w}_n)$ exists, where $B(\epsilon) = \{\boldsymbol{w}_n : \|\boldsymbol{w}_n - \boldsymbol{w}_n^\diamond\| < \epsilon\}$, and $\delta := Q^*(\boldsymbol{w}_n^\diamond) - \sup_{\boldsymbol{w}_n \in \mathcal{W}_n \setminus B(\epsilon)} Q^*(\boldsymbol{w}_n) > 0$.*

Assumption 2 restricts the shape of $Q^*(\boldsymbol{w}_n)$ around the global maximizer, which cannot be discontinuous or too flat. Given nonidentifiability of the neural network model, see e.g. Sun et al. (2022), we have implicitly assumed that each $\boldsymbol{w}_n$ is unique up to loss-invariant transformations, e.g., reordering the hidden neurons within the same hidden layer or simultaneously altering the signs of certain weights and biases. The same assumption has often been used in theoretical studies of neural networks, see e.g. Liang et al. (2018b).

On the other hand, by Theorem 1 of Liang et al. (2018a), we have

$$\sup_{\boldsymbol{w}_n \in \mathcal{W}_n} \left| \widehat{\mathcal{G}}(\boldsymbol{w}_n|\tilde{\boldsymbol{w}}_n^*) - \widetilde{\mathcal{G}}(\boldsymbol{w}_n|\tilde{\boldsymbol{w}}_n^*) \right| \xrightarrow{p} 0, \quad \text{as } n \to \infty, \tag{S4}$$

under some regularity conditions. Under Assumptions 1-2, Liang et al. (2024) proved the following lemma:

Lemma S1 *(Lemma 4.1; Liang et al. (2024)) Suppose Assumptions 1-2 hold, and the joint likelihood function* $\pi(\boldsymbol{Y}_n, \boldsymbol{Z}_n|\boldsymbol{X}_n, \boldsymbol{w}_n)$ *is continuous in* $\boldsymbol{w}_n$. *If* $\hat{\boldsymbol{w}}_n^*$ *is unique, then* $\boldsymbol{w}_n^*$ *that maximizes* $\pi(\boldsymbol{w}_n|\boldsymbol{X}_n, \boldsymbol{Y}_n)$ *as well as minimizes* $D_{KL}(\boldsymbol{w}_n)$ *is unique and, subsequently,* $\|\hat{\boldsymbol{w}}_n^* - \boldsymbol{w}_n^*\| \xrightarrow{p} 0$ *holds as* $n \to \infty$.

For Lemma S1, the uniqueness of $\hat{\boldsymbol{w}}_n^*$, up to some loss-invariant transformations as discussed previously, can be ensured by its consistency as established in the followed sections of this supplement, see equation (S20). The condition minimizing $D_{KL}(\boldsymbol{w}_n)$ is generally implied by $\tilde{U}_n(\boldsymbol{Y}_n, \boldsymbol{X}_n, \boldsymbol{Z}_n, \boldsymbol{w}_n) = 0$ provided the consistency of $\bar{\boldsymbol{\theta}}_n^*$, while the convergence of $\boldsymbol{w}_n^*$ to a maximizer of $\pi(\boldsymbol{w}_n|\boldsymbol{X}_n, \boldsymbol{Y}_n)$ is generally implied by the Monte Carlo nature of Algorithm 1. Therefore, by eq. (17) of the main text, if $\boldsymbol{w}_n^{(k)}$ converges and $\tilde{U}_n(\boldsymbol{Y}_n, \boldsymbol{X}_n, \boldsymbol{Z}_n, \boldsymbol{w}_n^{(k)})$ converges to 0, we would have $\|\hat{\boldsymbol{w}}_n^* - \boldsymbol{w}_n^*\| \xrightarrow{p} 0$ provided that the prior has been appropriately chosen such that $\hat{\boldsymbol{w}}_n^*$ is consistent for $\tilde{\boldsymbol{w}}_n^*$ and $\hat{g}(y_i, x_i, z_i, \hat{\boldsymbol{w}}_n^*)$ is consistent for $\boldsymbol{\theta}^*$.

In summary, by Lemma S1, if we can choose an appropriate prior $\pi(\boldsymbol{w})$ such that $\hat{\boldsymbol{w}}_n^*$ is consistent for $\tilde{\boldsymbol{w}}_n^*$, then $\boldsymbol{w}_n^*$ is also consistent for $\tilde{\boldsymbol{w}}_n^*$ and $\hat{g}(y, \boldsymbol{x}, z, \boldsymbol{w}_n^*)$ is consistent for $\boldsymbol{\theta}^*$. Establishing the consistency of $\hat{\boldsymbol{w}}_n^*$ will be the focus of Section §1.2 of this supplement. Note that showing the consistency of $\hat{\boldsymbol{w}}_n^*$ is relatively simpler than directly working on $\boldsymbol{w}_n^*$, as $\hat{\boldsymbol{w}}_n^*$ is based on the complete data.

Then, following from the consistency of $\hat{g}(\cdot)$, we immediately have

$$\|\frac{1}{n}\sum_{i=1}^{n} \hat{g}(y_i, x_i, z_i, \boldsymbol{w}_n^*) - \boldsymbol{\theta}^*\| \xrightarrow{p} 0, \quad \text{as } n \to \infty. \tag{S5}$$

As a slight relaxation for the definition of $G(\cdot)$, we can write equation (7) of the main text as

$$\boldsymbol{\theta}^* = \lim_{n\to\infty} G(\boldsymbol{Y}_n, \boldsymbol{X}_n, \boldsymbol{Z}_n), \tag{S6}$$

where $\boldsymbol{Z}_n$ is assumed to be known. By combining (S5) and (S6), we have

$$\left\|\frac{1}{n}\sum_{i=1}^{n} \hat{g}(y_i, x_i, z_i, \boldsymbol{w}_n^*) - G(\boldsymbol{Y}_n, \boldsymbol{X}_n, \boldsymbol{Z}_n)\right\| \xrightarrow{p} 0, \quad \text{as } n \to \infty,$$

i.e., the EFI estimator $\bar{\boldsymbol{\theta}}^* := \frac{1}{n}\sum_{i=1}^{n} \hat{g}(y_i, x_i, z_i, \boldsymbol{w}_n^*)$ is consistent for the inverse mapping $G(\boldsymbol{Y}_n, \boldsymbol{X}_n, \boldsymbol{Z}_n)$. Further, by Slutsky's theorem, the uncertainty of $\boldsymbol{Z}_n$ can be propagated to $\boldsymbol{\theta}$ via the EFI estimator. Therefore, the extended fiducial density (EFD) function of $\boldsymbol{\theta}$ can be approximated by

$$\tilde{\mu}_n(d\boldsymbol{\theta}) = \frac{1}{\mathcal{M}}\sum_{k=1}^{\mathcal{M}} \delta_{\bar{\boldsymbol{\theta}}^{*,k}}(d\boldsymbol{\theta}), \quad \text{as } \mathcal{M} \to \infty, \tag{S7}$$

where δ_a stands for the Dirac measure at a given point a, $\bar{\boldsymbol{\theta}}^{*,k} := \frac{1}{n}\sum_{i=1}^{n} \hat{g}(x_i, y_i, z_i^{*,k}, \boldsymbol{w}_n^*)$, and $\boldsymbol{Z}_n^{*,k} := (z_1^{*,k}, z_2^{*,k}, \ldots, z_n^{*,k})$ for $k = 1, 2, \ldots, \mathcal{M}$ denotes $\mathcal{M}$ random draws from the distribution $\pi(\boldsymbol{Z}_n|\boldsymbol{X}_n, \boldsymbol{Y}_n, \boldsymbol{w}_n^*)$ under the limit setting of ϵ.

§1.2 On the Consistency of $\hat{g}(\cdot)$ under the Large Model Scenario

In this section, we first show that $\hat{\boldsymbol{w}}_n^*$ is consistent under the framework of imputation-regularized optimization (IRO) algorithm (Liang et al., 2018a) by assuming that the true values of $\boldsymbol{Z}_n$ are known and $\boldsymbol{w}_n$ is subject to a mixture Gaussian prior. Subsequently, we show that $\hat{g}(\cdot)$ is consistent.

§1.2.1 An Auxiliary Stochastic Neural Network Model

To show $\hat{\boldsymbol{w}}_n^*$ is consistent, we introduce an auxiliary stochastic neural network (StoNet) model. For each of the hidden and output neurons of the model, we introduce a random noise:

$$\begin{aligned} \tilde{u}_{l,i} &= \sum_{j=0}^{d_{l-1}} w_{n,l,i,j} u_{l-1,j} + e_{l,i} := v_{l,i} + e_{l,i}, \\ u_{l,i} &= \Psi_l(\tilde{u}_{l,i}), \end{aligned} \tag{S8}$$

where $l \in \{1, 2, \ldots, H\}$ indexes the layers of the DNN, and $i \in \{1, 2, \ldots, d_l\}$ indexes the neurons at layer l of the DNN, the random noise $e_{l,i} \sim N(0, \sigma_l^2)$, $w_{n,l.i,j}$ denotes the weight on the connection from neuron i of layer l to neuron j of layer $l-1$, and $\Psi_l(\cdot)$ denotes the activation function used for layer l. Note that σ_l^2's are all known and pre-specified by the user, and $l = 0$ represents the input layer. As a consequence of introducing the random noise, we can treat $\tilde{U}_l$'s as latent variables, where $\tilde{U}_l = (\tilde{u}_{l,1}, \tilde{u}_{l,2}, \ldots, \tilde{u}_{l,d_l})^\top$.

When considering a dataset of size n, we let $\tilde{u}_{l,i,(k)}$ denote the latent variable imputed for neuron i of layer l for observation $k \in \{1, 2, \ldots, n\}$, let $\tilde{U}_{l,(k)} = (\tilde{u}_{l,1,(k)}, \tilde{u}_{l,2,(k)}, \ldots, \tilde{u}_{l,d_l,(k)})^\top$, and let $\tilde{\boldsymbol{U}}_l = \{\tilde{U}_{l,(1)}, \tilde{U}_{l,(2)}, \ldots, \tilde{U}_{l,(n)}\}$ denote the latent variables at layer l for all n observations. Recall that we have defined $\boldsymbol{Y}_n = \{y_1, y_2, \ldots, y_n\}$, $\boldsymbol{X}_n = \{\boldsymbol{x}_1, \boldsymbol{x}_2, \ldots, \boldsymbol{x}_n\}$, and $\boldsymbol{Z}_n = \{z_1, z_2, \ldots, z_n\}$. Given a set of pseudo-complete data $(\boldsymbol{Y}_n, \tilde{\boldsymbol{U}}_H, \ldots, \tilde{\boldsymbol{U}}_1, \boldsymbol{X}_n, \boldsymbol{Z}_n)$, we define the posterior distribution of $\boldsymbol{w}_n$ as:

$$\pi_\epsilon(\boldsymbol{w}_n | \boldsymbol{Y}_n, \tilde{\boldsymbol{U}}_H, \ldots, \tilde{\boldsymbol{U}}_1, \boldsymbol{X}_n, \boldsymbol{Z}_n) \propto \pi(\boldsymbol{w}_n) \prod_{k=1}^n p_\epsilon(y_k | \boldsymbol{x}_k, z_k, \bar{\boldsymbol{\theta}}) \prod_{l=1}^H \prod_{k=1}^n \pi_l(\tilde{U}_{l,(k)} | \tilde{U}_{l-1,(k)}, \boldsymbol{w}_{n,l}), \tag{S9}$$

where $\boldsymbol{w}_n = \{\boldsymbol{w}_{n,1}, \boldsymbol{w}_{n,2}, \ldots, \boldsymbol{w}_{n,H}\}$ with $\boldsymbol{w}_{n,l}$ being the connection weights for layer l,

$$p_\epsilon(y_k | \boldsymbol{x}_k, z_k, \bar{\boldsymbol{\theta}}) \propto \exp\{-(d(y_k, x_k, z_k, \bar{\boldsymbol{\theta}}) + \eta \|\hat{\boldsymbol{\theta}}_k - \bar{\boldsymbol{\theta}}\|^2)/\epsilon\},$$

$\tilde{\boldsymbol{U}}_{0,(k)} = \{y_k, \boldsymbol{x}_k, z_k\}$, $\hat{\boldsymbol{\theta}}_k = \tilde{U}_{H,(k)}$, and $\pi_l(\cdot)$ represents a d_l-dimensional multivariate Gaussian distribution.

Remark S1 *It is interesting to point out that for the stochastic version of the EFI network, $(\boldsymbol{Y}_n, \tilde{\boldsymbol{U}}_H, \ldots, \tilde{\boldsymbol{U}}_1)$ forms a directed cyclic graph (DCG): $\boldsymbol{Y}_n \to \tilde{\boldsymbol{U}}_1 \to \cdots \to \tilde{\boldsymbol{U}}_H \to \boldsymbol{Y}_n$. See e.g. Sethuraman et al. (2023) for analysis of DCGs. For our case, $\boldsymbol{Y}_n$ is known, which serves as intervention variables and greatly simplifies the problem. Specifically, as $\epsilon \downarrow 0$, $\hat{\boldsymbol{\theta}}_k$'s can be uniquely determined via $p_\epsilon(\cdot)$ for a given set of $(\boldsymbol{Y}_n, \boldsymbol{X}_n, \boldsymbol{Z}_n)$ and, therefore, (S9) is reduced to*

the posterior distribution for a conventional StoNet with $(y_k, \boldsymbol{x}_k, z_k)$ serving as the input and $\hat{\boldsymbol{\theta}}_k$ serving as the target output.

Assumption 3 *(i) The activation function $\Psi_l(\cdot)$ used for each hidden neuron is c'-Lipschitz continuous for some constant c'; (ii) $d_l \log(d_l) \prec n/\log(n)$ for $l = 0, 1, 2, \ldots, H$, where d_0 denotes the dimension of (Y, X, Z); (iii) the network's depth H and widths d_l's, for $l = 0, 1, 2, \ldots, H$, are all allowed to increase with the sample size n.*

Assumption 3-(i) has covered many commonly used activation functions such as *ReLU*, *tanh*, and *sigmoid*. Assumption 3-(ii) restricts the width of the DNN. To model a dataset of dimension d_0, we generally need a model of dimension $dim(\boldsymbol{\theta}) \succeq d_0$. Since we do not aim to impose any sparsity constraints on $\boldsymbol{\theta}$ in our main theory, we assume d_0 also satisfies the constraint $d_0 \log(d_0) \prec n$ such that $dim(\boldsymbol{\theta}) \prec n$ is still possible to hold.

Suppose Assumption 1 and Assumption 3 hold. Following Liang et al. (2022) and Sun and Liang (2022), we can establish the existence of a small value $\tau(d_1, d_2, \ldots, d_H)$, as a function of $d_1, d_2, \ldots, d_H$, such that if $\max\{\sigma_1, \sigma_2, \ldots, \sigma_H\} \prec \tau(d_1, d_2, \ldots, d_H)$ and $\epsilon \to \infty$, then

$$\sup_{\boldsymbol{w}_n \in \mathcal{W}_n} \frac{1}{n} \Big| \log \pi_\epsilon(\boldsymbol{w}_n | \boldsymbol{Y}_n, \tilde{\boldsymbol{U}}_H, \ldots, \tilde{\boldsymbol{U}}_1, \boldsymbol{X}_n, \boldsymbol{Z}_n) - \log \pi_\epsilon(\boldsymbol{w}_n | \boldsymbol{Y}_n, \boldsymbol{X}_n, \boldsymbol{Z}_n) \Big| \overset{p}{\to} 0, \quad \text{as } n \to \infty. \tag{S10}$$

In other words, the DNN model in the EFI network and stochastic DNN model introduced above have asymptotically the same loss function as long as $\sigma_1, \ldots, \sigma_H$ are sufficiently small and ϵ is sufficiently small.

Suppose that we want to estimate $\boldsymbol{w}_n$ by maximizing the posterior distribution of the pseudo-complete data, i.e.,

$$\hat{\boldsymbol{w}}_n^u = \arg\max_{\boldsymbol{w}_n} \Big\{ \log \pi_\epsilon(\boldsymbol{w}_n | \boldsymbol{Y}_n, \tilde{\boldsymbol{U}}_H, \ldots, \tilde{\boldsymbol{U}}_1, \boldsymbol{X}_n, \boldsymbol{Z}_n) \Big\}. \tag{S11}$$

In the next subsection, we establish the consistency of $\hat{\boldsymbol{w}}_n^u$, as an estimator of $\tilde{\boldsymbol{w}}_n^*$, under appropriate conditions. Note that by Assumptions 1-2 and (S10), we have

$$\|\hat{\boldsymbol{w}}_n^u - \hat{\boldsymbol{w}}_n^*\| \overset{p}{\to} 0, \quad \text{as } n \to \infty. \tag{S12}$$

§1.2.2 Consistency of the Sparse DNN Model Estimation

This section gives a constructive proof for the consistency of $\hat{\boldsymbol{w}}_n^u$ based on the IRO algorithm (Liang et al., 2018a). To solve the optimization problem in (S11), the IRO algorithm starts with an initial weight setting $\hat{\boldsymbol{w}}_n^{(0)}$ and then iterates between the *imputation* and *regularized-optimization* steps:

- **Imputation:** For each block, conditioned on the current parameter estimate $\hat{\boldsymbol{w}}_n^{(t)}$, simulate the latent variables $(\tilde{\boldsymbol{U}}_H^{(t+1)}, \ldots, \tilde{\boldsymbol{U}}_1^{(t+1)})$ from the predictive distribution

$$\begin{aligned}\pi(\tilde{\boldsymbol{U}}_H^{(t+1)}, \ldots, \tilde{\boldsymbol{U}}_1^{(t+1)} | \boldsymbol{Y}_n, \boldsymbol{X}_n, \boldsymbol{Z}_n, \hat{\boldsymbol{w}}_n^{(t)}) \propto & \prod_{k=1}^n p_\epsilon(y_k | \boldsymbol{x}_k, z_k, \bar{\boldsymbol{\theta}}^{(t+1)}) \\ & \times \prod_{l=1}^H \prod_{k=1}^n \pi_l(\tilde{U}_{l,(k)}^{(t+1)} | \tilde{U}_{l-1,(k)}^{(t+1)}, \hat{\boldsymbol{w}}_{n,l}^{(t)}),\end{aligned} \tag{S13}$$

where t indexes iterations, $\bar{\boldsymbol{\theta}}^{(t+1)} = \frac{1}{n}\sum_{k=1}^{n} \tilde{U}_{H,(k)}^{(t+1)}$, and $\hat{\boldsymbol{w}}_{n,l}^{(t)}$ denotes the component of $\hat{\boldsymbol{w}}_n^{(t)}$ corresponding to the weights at the l-th layer.

- **Regularized-optimization:** Given the pseudo-complete data $\{\tilde{\boldsymbol{U}}_H^{(t+1)}, \ldots, \tilde{\boldsymbol{U}}_1^{(t+1)}, \boldsymbol{Y}_n, \boldsymbol{X}_n, \boldsymbol{Z}_n\}$, update $\hat{\boldsymbol{w}}_n^{(t)}$ by maximizing the penalized log-likelihood function:

$$\hat{\boldsymbol{w}}_n^{(t+1)} = \arg\max_{\boldsymbol{w}_n} \Big\{ \log \pi(\boldsymbol{Y}_n, \tilde{\boldsymbol{U}}_H^{(t+1)}, \ldots, \tilde{\boldsymbol{U}}_1^{(t+1)} | \boldsymbol{X}_n, \boldsymbol{Z}_n, \boldsymbol{w}_n) + \log \pi(\boldsymbol{w}_n) \Big\}, \tag{S14}$$

which, by the decomposition (S9), can be reduced to solving for $\hat{\boldsymbol{w}}_{n,1}^{(t+1)}, \ldots, \hat{\boldsymbol{w}}_{n,H}^{(t+1)}$, separately. The penalty function $\log \pi(\boldsymbol{w}_n)$ should be chosen such that $\hat{\boldsymbol{w}}_n^{(t+1)}$ forms a consistent estimator for the working true parameter

$$\begin{aligned} \boldsymbol{w}_{n,*}^{(t+1)} &= \arg\max_{\boldsymbol{w}_n} \mathbb{E}_{\hat{\boldsymbol{w}}_n^{(t)}} \log \pi(\boldsymbol{Y}_n, \tilde{\boldsymbol{U}}_H^{(t+1)}, \ldots, \tilde{\boldsymbol{U}}_1^{(t+1)} | \boldsymbol{X}_n, \boldsymbol{Z}_n, \boldsymbol{w}_n) \\ &= \arg\max_{\boldsymbol{w}_n} \int \log \pi(\boldsymbol{Y}_n, \tilde{\boldsymbol{U}}_H^{(t+1)}, \ldots, \tilde{\boldsymbol{U}}_1^{(t+1)} | \boldsymbol{X}_n, \boldsymbol{Z}_n, \boldsymbol{w}_n) \\ &\quad \times \pi(\tilde{\boldsymbol{U}}_H^{(t+1)}, \ldots, \tilde{\boldsymbol{U}}_1^{(t+1)} | \boldsymbol{Y}_n, \boldsymbol{X}_n, \boldsymbol{Z}_n, \hat{\boldsymbol{w}}_n^{(t)}) \pi_\epsilon(\boldsymbol{Y}_n | \boldsymbol{X}_n, \boldsymbol{Z}_n, \tilde{\boldsymbol{w}}_n^*) d\tilde{\boldsymbol{U}}_H^{(t+1)} \cdots d\tilde{\boldsymbol{U}}_1^{(t+1)} d\boldsymbol{Y}_n, \end{aligned} \tag{S15}$$

where the likelihood function of the latent variables $(\tilde{\boldsymbol{U}}_H^{(t+1)}, \ldots, \tilde{\boldsymbol{U}}_1^{(t+1)})$ is evaluated at $\hat{\boldsymbol{w}}_n^{(t)}$, and $\tilde{\boldsymbol{w}}_n^*$ corresponds to the true parameters of the underlying sparse DNN model.

To prove the consistency of $\hat{\boldsymbol{w}}_n^{(t)}$ as n and t approach to infinity, we need Assumptions 4-6 as specified below. For a matrix $\boldsymbol{\Sigma}$, we define the m-sparse minimal eigenvalues as follows:

$$\phi_{\min}(m|\boldsymbol{\Sigma}) = \min_{\boldsymbol{\beta}: \|\boldsymbol{\beta}\|_0 \le m} \frac{\boldsymbol{\beta}^\top \boldsymbol{\Sigma} \boldsymbol{\beta}}{\boldsymbol{\beta}^\top \boldsymbol{\beta}},$$

which represents the minimal eigenvalues of any $m \times m$-dimensional principal submatrix, and $\|\boldsymbol{\beta}\|_0$ denotes the number of nonzero elements in $\boldsymbol{\beta}$. Let $\boldsymbol{\Sigma}_{l,i}^{(t)} \in \mathbb{R}^{d_{l-1} \times d_{l-1}}$ denote the sample covariance matrix of the input variables for the regression formed for neuron i of layer l at iteration t, and let $s_{l,i}^{(t)}$ denote the size of the true regression model as implied by the working true parameter $\boldsymbol{w}_{n,*}^{(t)}$.

Assumption 4 *(i) All the variables $\{\boldsymbol{X}_n, \boldsymbol{Y}_n, \boldsymbol{Z}_n^{(t)}\}$ are uniformly bounded, where $\boldsymbol{Z}_n^{(t)}$ denotes the imputed values of $\boldsymbol{Z}_n$ at iteration t; (ii) there exist some constants $\kappa_{0,1}$ and $s_{1,i}^{(t)} \le \tilde{s}_{1,i}^{(t)} \le \min\{d_0, n\}$ such that $\phi_{\min}(\tilde{s}_{1,i}^{(t)}|\boldsymbol{\Sigma}_{1,i}^{(t)}) \ge \kappa_{0,1}$ holds uniformly for any neuron $i \in \{1, 2, \ldots, d_1\}$ and any iteration $t \in \{1, 2, \ldots, T\}$, where $\boldsymbol{\Sigma}_{1,i}^{(t)}$ denotes the covariance matrix of $\{\boldsymbol{X}_n, \boldsymbol{Y}_n, \boldsymbol{Z}_n^{(t)}\}$.*

Assumption 4-(i) restricts the pseudo-complete data $(\boldsymbol{x}, \boldsymbol{y}, \boldsymbol{z})$ to be uniformly bounded. To satisfy this condition, we can add a data transformation/normalization layer to the DNN model, ensuring that the transformed input values fall within the bounded set. Specifically, the transformation/normalization layer can form a bijective mapping and contain no tuning parameters. For example, when dealing with standard Gaussian random variables, we can transform them to be uniform over (0,1) via the probability integral transformation $\Phi(\cdot)$, the cumulative distribution function (CDF) of the standard Gaussian random variable.

Assumption 4-(ii) is natural for the problem. As implied by Assumption 3-(ii), the upper bound $s_{1,i}^{(t)} \leq \tilde{s}_{1,i}^{(t)} \leq \min\{d_0, n\}$ can always hold. For other layers, the upper bound is also true by Assumption 3-(ii), and the sparse eigenvalue property can be directly established, see Lemma S3 below.

Lemma S2 *Consider a random matrix $\boldsymbol{U} \in \mathbb{R}^{n \times d}$ with $n \geq d$. Suppose that the eigenvalues of $\boldsymbol{U}^\top \boldsymbol{U}$ are upper bounded, i.e., $\lambda_{\max}(\boldsymbol{U}^\top \boldsymbol{U}) \leq n\kappa_{\max}$ for some constant $\kappa_{\max} > 0$. Let $\Psi(\boldsymbol{U})$ denote an elementwise transformation of $\boldsymbol{U}$. Then*

$$\lambda_{\max}\left((\Psi(\boldsymbol{U}))^\top (\Psi(\boldsymbol{U}))\right) \leq n\kappa_{\max}, \tag{S16}$$

for the tanh, sigmoid and ReLU transformations.

PROOF: For ReLU, (S16) follows from Lemma 5 of Dittmer et al. (2018). For *tanh* and *sigmoid*, since they are Lipschitz continuous with a Lipschitz constant of 1, Lemma 5 of Dittmer et al. (2018) also applies. □

Lemma S3 *Consider an auxiliary stochastic neural network as defined in (S8) with an activation function tanh, sigmoid, or ReLU. Then for any any layer $l \in \{2, 3, \ldots, H\}$, neuron $i \in \{1, 2, \ldots, d_l\}$, and iteration $t \in \{1, 2, \ldots, T\}$, there exists a number $\tilde{s}_{l,i}^{(t)}$ such that $s_{l,i}^{(t)} \leq \tilde{s}_{l,i}^{(t)} \leq \min\{d_{l-1}, n\}$ and $\phi_{\min}(\tilde{s}_{l,i}^{(t)} | \boldsymbol{\Sigma}_{l,i}^{(t)}) \geq \kappa_{0,l}$ hold.*

PROOF: We use $\tilde{U}_l \in \mathbb{R}^{d_l}$ and $V_l \in \mathbb{R}^{d_l}$ to denote generic vectors corresponding to the l-th layer, where $\tilde{U}_k = V_l + \boldsymbol{e}_l$. Additionally, we use $\tilde{\boldsymbol{U}}_l \in \mathbb{R}^{n \times d_l}$ and $\boldsymbol{V}_l \in \mathbb{R}^{n \times d_l}$ to denote the matrices (for all observations) corresponding to the l-th layer. For the auxiliary stochastic neural network, since σ_l^2's have been set to very small values, it follows from (S8) that

$$\Psi(\tilde{U}_l) \approx \Psi(V_l) + \nabla_{V_l}\Psi(V_l) \circ \boldsymbol{e}_l, \quad l = 1, 2, \ldots, H-1,$$

where $\circ$ denotes elementwise product, $V_l = (v_{l,1}, v_{l,2}, \ldots, v_{l,d_l})^\top$, and $\boldsymbol{e}_l = (e_{l,1}, e_{l,2}, \ldots, e_{l,d_l})^\top$. Then, for any $i \in \{1, 2, \ldots, d_{l+1}\}$,

$$\begin{aligned} \boldsymbol{\Sigma}_{l+1,i} &\approx \mathrm{Var}(\mathbb{E}(\Psi(V_l) + \nabla_{V_l}\Psi(V_l) \circ \boldsymbol{e}_l | V_l)) + \mathbb{E}(\mathrm{Var}(\Psi(V_l) + \nabla_{V_l}\Psi(V_l) \circ \boldsymbol{e}_l | V_l)) \\ &= \mathrm{Var}(\Psi(V_l)) + \mathrm{Diag}\left\{\sigma_l^2 \mathbb{E}[\nabla_{V_l}\Psi(V_l) \circ \nabla_{V_l}\Psi(V_l)]\right\}, \end{aligned} \tag{S17}$$

where $\mathrm{Diag}\{\boldsymbol{a}\}$, $\boldsymbol{a} \in \mathbb{R}^{d_l}$, denotes a $d_l \times d_l$ diagonal matrix with the diagonal elements given by the vector $\boldsymbol{a}$.

By induction, building on Assumption 4-(i) and Lemma S2, we can assume that the maximum eigenvalue of $(\Psi(\tilde{\boldsymbol{U}}_{l-1}))^\top \Psi(\tilde{\boldsymbol{U}}_{l-1})$ is upper bounded by $n\kappa_{\max}$. By an extension of Ostrowski's theorem, see Theorem 3.2 of Higham and Cheng (1998), we have

$$\begin{aligned} \lambda_{\max}(\boldsymbol{V}_l^T \boldsymbol{V}_l) &= \max_{\|\boldsymbol{u}\|=1} \boldsymbol{u}^\top \boldsymbol{w}_{n,l} (\Psi(\tilde{\boldsymbol{U}}_{l-1}))^\top \Psi(\tilde{\boldsymbol{U}}_{l-1}) \boldsymbol{w}_{n,l}^\top \boldsymbol{u} \\ &\leq \lambda_{\max}((\Psi(\tilde{\boldsymbol{U}}_{l-1}))^\top \Psi(\tilde{\boldsymbol{U}}_{l-1})) \max_{\|\boldsymbol{u}\|=1} \boldsymbol{u}^\top \boldsymbol{w}_{n,l} \boldsymbol{w}_{n,l}^\top \boldsymbol{u} \\ &= n\kappa_{\max}\tau_{\max}, \end{aligned}$$

where the existence of the upper bound $\lambda_{\max}(\boldsymbol{w}_{n,l}^\top \boldsymbol{w}_{n,l}) \leq \tau_{\max}$ follows from the boundedness of $\boldsymbol{w}_n$ as implied by Assumption 1-(i). By choosing $\boldsymbol{u}$ as a one-hot vector, it is easy to see that for any $i \in \{1, 2, \dots, d_l\}$,

$$\sum_{j=1}^{n} V_{l,i,j}^2 \leq \lambda_{\max}(\boldsymbol{V}_l^\top \boldsymbol{V}_l) \leq n\kappa_{\max}\tau_{\max}, \tag{S18}$$

where $V_{l,i,j}$ denotes the (j,i)th element of $\boldsymbol{V}_l$. This further implies, as $n \to \infty$,

$$\mathbb{E}(V_{l,i,j}^2) \leq \kappa_{\max}\tau_{\max}. \tag{S19}$$

In words, $V_{l,i,j}$ has bounded mean and variance.

By Markov's inequality, we can bound $V_{l,i,j}$ to a closed interval with a high probability, i.e., $P(|V_{l,i,j}| \leq C) \geq 1 - \kappa_{\max}\tau_{\max}/C^2$ for some large constant C. Therefore, for any activation function which has nonzero gradients on any closed interval, e.g., *tanh* and *sigmoid*, there exists a constant $c > 0$ such that

$$\mathbb{E}[\nabla_{V_{l,i,j}} \Psi(V_{l,i,j})]^2 \geq c.$$

which implies $\phi_{\min}(d_l|\boldsymbol{\Sigma}_l) \geq c\sigma_l^2$.

For the ReLU activation function, if a hidden neuron belongs to the true neuron set at iteration t (as determined by $\boldsymbol{w}_{n,*}^{(t)}$), then $\Psi(V_{l,i,j}^{(t)})$ cannot be constantly 0 over all n samples. Therefore, it is reasonable to assume that there exists a threshold $q_{\min} \in (0,1)$ such that $\mathbb{E}[\nabla_{V_{l,i,j}} \Psi(V_{l,i,j}^{(t)})]^2 \geq q_{\min}$ for any true neuron in all iterations. Under this assumption, we would at least have

$$\phi_{\min}(|\boldsymbol{s}_l^{(t)}||\boldsymbol{\Sigma}_l) \geq \sigma_l^2 q_{\min}, \quad l = 1, 2, \dots, h; \quad t = 1, 2, \dots, T,$$

where $\boldsymbol{s}_l^{(t)} \subset \boldsymbol{S}^{(t)}$ denotes the set of true neurons at layer l. □

To study the property of the coefficient estimator for each regression formed in the auxiliary stochastic neural network, we introduce the following lemma, which is a restatement of Theorem 3.4 of Song and Liang (2023).

Lemma S4 *(Theorem 3.4; Song and Liang (2023)) Consider a linear regression*

$$\boldsymbol{y} = \boldsymbol{X}\boldsymbol{\beta} + \sigma\boldsymbol{\epsilon},$$

where $\boldsymbol{y} \in \mathbb{R}^n$, $\boldsymbol{X} \in \mathbb{R}^{n \times p_n}$, $\boldsymbol{\beta} \in \mathbb{R}^{p_n}$, $\sigma > 0$, *and* $\boldsymbol{\epsilon} \sim \mathcal{N}(0, I_{p_n})$ *is Gaussian noise. Suppose that the model satisfies the following conditions:*

(A1) (i) All the covariates are uniformly bounded; (ii) the dimensionality can be high with $p_n \geq n$; *and (iii) there exists some integer* $\bar{p}$ *(depending on* n *and* p_n*) and a fixed constant* κ_0 *such that* $\bar{p} \succ s_n$ *and* $\lambda_{\min}(\boldsymbol{X}_\xi^\top \boldsymbol{X}_\xi) \geq n\kappa_0$ *for any subset model* $|\xi| \leq \bar{p}$, *where* s_n *denotes the size of the true model* ξ^*, *and* $\lambda_{\min}(\cdot)$ *denotes the minimum eigenvalue of a square matrix. Let* $\hat{\boldsymbol{\beta}}_\xi$ *denote the MLE of the true model.*

(A2) (i) $s_n \log p_n \prec n$*; (ii)* $\max\{|\beta_j^*/\sigma^*| : j = 1, 2, \ldots, p_n\} \leq \gamma_3 E_n$*, where* β_j^**'s and* σ^* *denote the true parameter values of the regression model,* $\gamma_3 \in (0, 1)$ *is a fixed constant, and* E_n *is nondecreasing with respect to* n*.*

(A3) Let each component of $\boldsymbol{\beta}$ *be subject to the following mixture Gaussian prior distribution*

$$\beta_j/\sigma^* \sim (1-\rho)\mathcal{N}(0, \tilde{\sigma}_0^2) + \rho\mathcal{N}(0, \tilde{\sigma}_1^2), \quad j = 1, 2, \ldots, p_n,$$

where $E_n/\tilde{\sigma}_1^2 + \log \tilde{\sigma}_1 \asymp \log p_n$*,* $\rho = 1/p_n^{1+u}$*, and* $\tilde{\sigma}_0 \leq a_n/\sqrt{2(1+u)\log p_n}$ *for some constant* $u > 1$ *and sequence* $a_n \prec \sqrt{1/(ns_n \log p_n)}/p_n$*. Additionally,* $s_n E_n \sqrt{s_n \log p_n/n} \prec \tilde{\sigma}_1^2$ *and* $\min_{j\in\xi^*} |\beta_j^*| \geq M_1\sqrt{\log p_n/n}$ *for some sufficiently large* $M_1 > 0$*.*

Let $\hat{\boldsymbol{\beta}}_n$ *denote the MAP estimator of* $\boldsymbol{\beta}$*. Then there exists a constant* c *such that*

$$\mathbb{E}\|\hat{\boldsymbol{\beta}}_n - \boldsymbol{\beta}^*\|^2 = c(\sigma^*)^2\|\boldsymbol{X}_{\xi^*}^\top \boldsymbol{X}_{\xi^*}\|^{-1} \leq \frac{c(\sigma^*)^2}{n\kappa_0},$$

Specifically, with dominating probability, we have $\hat{\boldsymbol{\beta}}_{n,j} = \hat{\boldsymbol{\beta}}_{\xi^*,j'}$ *if* $j \in \xi^*$ *and 0 otherwise, where* $\hat{\boldsymbol{\beta}}_{n,j}$ *denotes the* j*th element of* $\hat{\boldsymbol{\beta}}_n$*, and* $\hat{\boldsymbol{\beta}}_{\xi^*,j'}$ *denotes the element of* $\hat{\boldsymbol{\beta}}_{\xi^*}$ *that corresponds to* $\boldsymbol{\beta}_{n,j}$*.*

It is easy to see that under Assumption 1, Assumption 3, and Assumption 4, each linear regression formed in the stochastic neural network satisfies conditions (A1) and (A2) of Lemma S4. In particular, we can set E_n as the diameter of $\mathcal{W}_n$. To ensure the condition (A3) to be satisfied, we make the following assumption:

Assumption 5 *Let each connection weight* $w_{n,l,i,j}$ *be subject to the following mixture Gaussian priro distribution:*

$$w_{n,l,i,j}/\sigma_l \sim (1-\rho)\mathcal{N}(0, \tilde{\sigma}_{0,l}^2) + \rho\mathcal{N}(0, \tilde{\sigma}_{1,l}^2), \quad l = 1, 2, \ldots, H, \quad i = 1, 2, \ldots, d_l, \quad j = 1, 2, \ldots, d_{l-1},$$

where we set ρ*,* $\tilde{\sigma}_{0,l}$ *and* $\tilde{\sigma}_{1,l}$ *such that* $\rho = 1/d_{l-1}^{1+u}$*,* $\tilde{\sigma}_{0,l} \prec 1/(d_{l-1}^{3/2}\log(d_{l-1})\sqrt{2(1+u)n})$*, and* $E_n/\tilde{\sigma}_{1,l}^2 + \log \tilde{\sigma}_{1,l} \asymp \log d_{l-1}$ *for some constant* $u > 1$ *and any* $l = 1, 2, \ldots, H$*. Additionally, there exists some constant* $M_1 > 0$ *such that* $\min_{1\leq i\leq d_l, j\in\xi_i^*} |w_{n,l,i,j}^*| \geq M_1\sqrt{\log d_{l-1}/n}$*.*

It is easy to verify that the condition (A3) holds under Assumption 3-(ii) and Assumption 5. In addition, the w-min condition, i.e., $\min_{1\leq i\leq d_l, j\in\xi_i^*} |w_{n,l,i,j}^*| \geq M_1\sqrt{\log d_{l-1}/n}$, is rather weak and can be generally satisfied as n becomes large.

Theorem S1 *Suppose that a mixture Gaussian penalty is imposed on the weights of the DNN model in the EFI network and Assumptions 1 and 3-5 hold. Furthermore, suppose* $\sum_{l=1}^H d_l\sigma_l^2/\sigma_{l-1}^2 \prec n$ *holds, where* $\sigma_0 = O(1)$ *represents a constant. Then there exist a constant* c *such that*

$$E\|\hat{\boldsymbol{w}}_n^{(t)} - \boldsymbol{w}_{n,*}^{(t)}\|^2 \leq \frac{c}{n}\sum_{l=1}^H d_l\frac{\sigma_l^2}{\sigma_{l-1}^2} := r_n \prec o(1).$$

PROOF: By the above analysis, each regression formed in the stochastic neural network satisfies the conditions of Lemma S4. Therefore, the sparse eigenvalue lower bounds established in Lemma S3 hold for the stochastic neural network. Further, by summarizing the l_2-errors of coefficient estimation for all $\sum_{l=1}^{H} d_l$ linear regressions, we can conclude the proof. $\square$

It is important to note that the condition $\sum_{l=1}^{H} d_l \sigma_l^2/\sigma_{l-1}^2 \prec n$ allows the width of each layer of the neural network to increase with n at a rate as high as $O(n/\log(n))$. This accommodates the scenarios where $dim(\boldsymbol{\theta}) = O(n^{\zeta})$ for some $\frac{1}{2} \leq \zeta < 1$. In this case, we have $d_H = dim(\boldsymbol{\theta})$ for the DNN model in the EFI network, which enables the uncertainty of $\boldsymbol{\theta}$ to be properly quantified as implied by Theorem 3.1 proved below.

Further, let's consider the mapping $M(\boldsymbol{w}_n)$ as defined in (S15), i.e.,

$$M(\boldsymbol{w}_n) = \arg\max_{\boldsymbol{w}_n'} \mathbb{E}_{\boldsymbol{w}_n} \log \pi(\boldsymbol{Y}_n, \tilde{\boldsymbol{U}}_H, \ldots, \tilde{\boldsymbol{U}}_1 | \boldsymbol{X}_n, \boldsymbol{Z}_n, \boldsymbol{w}_n').$$

As argued in Liang et al. (2018a) and Nielsen (2000), it is reasonable to assume that the mapping is contractive. A recursive application of the mapping, i.e., setting $\hat{\boldsymbol{w}}_n^{(t+1)} = \boldsymbol{w}_{n,*}^{(t+1)} = M(\hat{\boldsymbol{w}}_n^{(t)})$, leads to a monotone increase of the target expectations

$$\mathbb{E}_{\hat{\boldsymbol{w}}_n^{(t)}} \log \pi(\boldsymbol{Y}_n, \tilde{\boldsymbol{U}}_H^{(t+1)}, \ldots, \tilde{\boldsymbol{U}}_1^{(t+1)} | \boldsymbol{X}_n, \boldsymbol{Z}_n, \hat{\boldsymbol{w}}_n^{(t+1)})$$

for $t = 1, 2, \ldots, T$.

Assumption 6 *The mapping $M(\boldsymbol{w}_n)$ is differentiable. Let $\lambda_{\max}(M_{\boldsymbol{w}_n})$ be the largest singular value of $\partial M(\boldsymbol{w}_n)/\partial \boldsymbol{w}_n$. There exists a number $\lambda^* < 1$ such that $\lambda_{\max}(M_{\boldsymbol{w}_n}) \leq \lambda^*$ for all $\boldsymbol{w}_n \in \mathcal{W}_n$ for sufficiently large n and almost every training dataset D_n.*

Theorem S2 *Suppose that a mixture Gaussian penalty is imposed on the weights of the DNN model in the EFI network; Assumptions 1 and 3-6 hold; ϵ is sufficiently small; and $\sum_{l=1}^{H} d_l \sigma_l^2/\sigma_{l-1}^2 \prec n$ holds, where $\max\{\sigma_1, \sigma_2, \ldots, \sigma_H\} \prec \tau(d_1, d_2, \ldots, d_H)$ as defined in Section §1.2.1 and $\sigma_0 = O(1)$ represents a constant. Then $\|\hat{\boldsymbol{w}}_n^{(t)} - \tilde{\boldsymbol{w}}_n^*\| \xrightarrow{p} 0$ for sufficiently large n and sufficiently large t and almost every training dataset D_n.*

PROOF: This lemma directly follows from Theorem 4 of Liang et al. (2018a) that the estimator $\hat{\boldsymbol{w}}_n^{(t)}$ is consistent when both n and t are sufficiently large. $\square$

In summary, we have given a constructive proof for the consistency of sparse stochastic neural network based on the sparse learning theory developed for high-dimensional linear regression. Our proof implies that under the conditions of Theorem S2, such a consistent estimator can also be obtained by a direct calculation of $\hat{\boldsymbol{w}}_n^u$ as defined in (S11). Furthermore, it follows from (S12) that

$$\|\hat{\boldsymbol{w}}_n^* - \tilde{\boldsymbol{w}}_n^*\| \xrightarrow{p} 0, \quad \text{as } n \to \infty. \tag{S20}$$

Proof of Theorem 3.1

Proof: As a summary of Lemma S1 and (S20), we have

$$\|\boldsymbol{w}_n^* - \tilde{\boldsymbol{w}}_n^*\| \xrightarrow{p} 0, \quad \text{as } n \to \infty,$$

by setting $\sigma_1 = \sigma_2 = \cdots = \sigma_H \prec \tau(d_1, d_2, \ldots, d_H)$. Subsequently, $\hat{g}(y, \boldsymbol{x}, z, \boldsymbol{w}_n^*)$ constitutes a consistent estimator of $\boldsymbol{\theta}$, and so does $G^*(\boldsymbol{Y}_n, \boldsymbol{X}_n, \boldsymbol{Z}_n)$ following the arguments in Section §1.1. □

In summary, through the introduction of an auxiliary stochastic neural network model and the utilization of the convergence theory of the IRO algorithm, we have justified the consistency of the sparse DNN model under mild conditions.

Remark S2 *The result presented in Theorem 3.1 is interesting: we can set the width of each layer of the DNN model to be of order $O(n^{\alpha})$ for some $1/2 \le \alpha < 1$ and set its depth to be $O(n^{\alpha'})$ for some $0 < \alpha' < 1 - \alpha$. Given the approximation ability of the sparse DNN model as studied in Sun et al. (2022), where it was proven that a neural network of size $O(n^{\tilde{\alpha}})$ for some $0 < \tilde{\alpha} < 1$ has been large enough for approximating many classes of functions, EFI can be used for uncertainty quantification for deep neural networks if their sizes are appropriately chosen. Specifically, for the Double-NN approach, we can set the size of the first neural network (for approximation of the inverse function $\boldsymbol{\theta} = G(\boldsymbol{Y}_n, \boldsymbol{X}_n, \boldsymbol{Z}_n)$) to be large under the constraint $\sum_{l=1}^{H} d_l \prec n$, and set the second neural network (for approximation of the function $\boldsymbol{Y}_n = f(\boldsymbol{X}_n, \boldsymbol{Z}_n; \boldsymbol{\theta})$) to be relatively small with the size $O(n^{\tilde{\alpha}})$ for some $0 < \tilde{\alpha} < 1$. By the theory developed in this paper, the uncertainty of the second neural network can still be correctly quantified using EFI.*

§2 CQR Method

For a test point $\boldsymbol{x}$ belonging to the class $\mathcal{I}_c$, CQR aims to find the intervals $\hat{C}_1(\boldsymbol{x})$ and $\hat{C}_{ITE}(\boldsymbol{x})$ such that

$$P(Y(1; \boldsymbol{x}) \in \hat{C}_1(\boldsymbol{x})) \ge 1 - \alpha, \quad P(Y(1; \boldsymbol{x}) - Y^{obs}(0; \boldsymbol{x}) \in \hat{C}_{ITE}(\boldsymbol{x})) \ge 1 - \alpha.$$

Similarly, for a test point $\boldsymbol{x}$ belonging to the class $\mathcal{I}_t$, CQR aims to find the intervals $\hat{C}_0(\boldsymbol{x})$ and $\hat{C}_{ITE}(\boldsymbol{x})$ such that

$$P(Y(0; \boldsymbol{x}) \in \hat{C}_0(\boldsymbol{x})) \ge 1 - \alpha, \quad P(Y^{obs}(1; \boldsymbol{x}) - Y(0; \boldsymbol{x}) \in \hat{C}_{ITE}(\boldsymbol{x})) \ge 1 - \alpha.$$

To achieve the above goals, CQR initially splits the training dataset $\mathcal{X}_{train}$ to $(\mathcal{X}_{train}, \mathcal{X}_{valid})$. A quantile regression model, such as BART or random forest, is trained on $\mathcal{X}_{train}$. Denote the output of the quantile regression by $[\hat{q}_{\alpha/2}(t, \boldsymbol{x}), \hat{q}_{1-\alpha/2}(t, \boldsymbol{x})]$, and calculate the score $s_i(t) = max(\hat{q}_{\alpha/2}(t, \boldsymbol{x}_i) - y_i(t), y_i(t) - \hat{q}_{1-\alpha/2}(t, \boldsymbol{x}_i))$ for each $\boldsymbol{x}_i \in \mathcal{X}_{valid}$. Note that s_i acts like a residual

derived from the validation set. Let $\hat{s}(t,\alpha) = Quantile(\{s_i(t)\}_{\boldsymbol{x}_i \in \mathcal{X}_{valid}}; [\frac{(n+1)\alpha]}{n}])$. Then $\hat{C}_t(\boldsymbol{x}_j)$ given below is the conformal prediction interval for any $\boldsymbol{x}_j \in \mathcal{X}_{test}$:

$$\begin{aligned}\hat{C}_t(\boldsymbol{x}_j) &= [\hat{q}_{\alpha/2}(t,\boldsymbol{x}_j) - \hat{s}(t,1-\alpha), \hat{q}_{1-\alpha/2}(t,\boldsymbol{x}_j) + \hat{s}(t,1-\alpha)] \\ &= [\hat{Y}^L(t;\boldsymbol{x}_j), \hat{Y}^R(t;\boldsymbol{x}_j)]\end{aligned}$$

For a more refined approach, one can consider incorporating propensity scores as weights as discussed in Tibshirani et al. (2019).

Case (i) and Case (ii) described in Section 5.1 can be addressed by the above approach. However, for Case (iii) there, the conformal prediction needs further steps. First, construct a pair of prediction intervals at level $1-\alpha/2$; namely, $[\hat{Y}^L(1;\boldsymbol{x}), \hat{Y}^R(1;\boldsymbol{x})]$ for $Y(1)$ and $[\hat{Y}^L(0;\boldsymbol{x}), \hat{Y}^R(0;\boldsymbol{x})]$ for $Y(0)$. Then, construct an interval for ITE as follows:

$$\hat{C}_{ITE}^{naive}(\boldsymbol{x}) = [\hat{Y}^L(1;\boldsymbol{x}) - \hat{Y}^R(0;\boldsymbol{x}), \hat{Y}^R(1;\boldsymbol{x}) - \hat{Y}^L(0;\boldsymbol{x})].$$

We refer to this approach as the "naive" approach, which usually leads to very wide prediction intervals.

Another option for case (iii) is the so-called "nested" approach by splitting $\mathcal{X}_{train}$ into two folds, denoted by $(\mathcal{X}_{train,1}, \mathcal{X}_{train,2})$. On the first fold, train $\hat{C}(1,\boldsymbol{x})$ and $\hat{C}(0,\boldsymbol{x})$ by applying conformal inference. On the second fold, for each $\boldsymbol{x}_i \in \mathcal{X}_{train,2}$, compute

$$\hat{C}(\boldsymbol{x}_i) = \begin{cases} [Y^{obs}(1;\boldsymbol{x}_i) - \hat{Y}^R(0;\boldsymbol{x}_i), Y^{obs}(1,\boldsymbol{x}_i) - \hat{Y}^L(0;\boldsymbol{x}_i)], & if\ \ T=1, \\ [\hat{Y}^L(1;\boldsymbol{x}_i) - Y^{obs}(0;\boldsymbol{x}_i), \hat{Y}^R(1;\boldsymbol{x}_i) - Y^{obs}(0;\boldsymbol{x}_i)], & if\ \ T=0, \end{cases}$$

where $\hat{Y}^L$ and $\hat{Y}^R$ are estimated based on $\mathcal{X}_{train,1}$. Note that the conformal inference is also applicable for the data with interval outcomes. Applying the conformal inference method with interval outcomes on $(\boldsymbol{x}_i, \hat{C}(\boldsymbol{x}_i))$ for $\boldsymbol{x}_i \in \mathcal{X}_{train,2}$, yielding the interval $\hat{C}_{ITE}^{exact}(\boldsymbol{x})$. Applying the conformal inference twice results in a sparse utilization of data for training the regression model and, subsequently, wider prediction intervals. The inexact method involves fitting conditional quantiles of $\hat{C}^L$ and $\hat{C}^R$, yielding an interval $\tilde{C}_{ITE}^{inexact}(\boldsymbol{x})$. The inexact method does not guarantee the coverage rate. Refer to Lei and Candès (2021) for the detail.

§3 Experimental Settings

To enforce a sparse DNN to be learned for the inverse function $g(\cdot)$, we impose the following mixture Gaussian prior on each element of $\boldsymbol{w}_n$:

$$\pi(w) \sim \rho N(0,\sigma_1^2) + (1-\rho) N(0,\sigma_0^2), \tag{S21}$$

where w represents a generic element of $\boldsymbol{w}_n$ and, unless stated otherwise, we set $\rho = 1e-2$, $\sigma_0 = 1e-2$ and $\sigma_1 = 1$. The elements of $\boldsymbol{w}_n$ are *a priori* independent.

For EFI, we employ SGHMC in latent variable sampling, i.e., we simulate $\boldsymbol{Z}_n^{(k+1)}$ in the following formula:

$$\begin{aligned}&\boldsymbol{V}_n^{(k+1)} = (1-\varpi)\boldsymbol{V}_n^{(k)} + \upsilon_{k+1}\widehat{\nabla}_{\boldsymbol{Z}_n} \log \pi_\epsilon(\boldsymbol{Z}_n^{(k)}|\boldsymbol{X}_n, \boldsymbol{Y}_n, \boldsymbol{w}_n^{(k)}) + \sqrt{2\varpi\tau\upsilon_{k+1}}\boldsymbol{e}^{(k+1)}, \\ &\boldsymbol{Z}_n^{(k+1)} = \boldsymbol{Z}_n^{(k)} + \boldsymbol{V}_n^{(k+1)},\end{aligned}$$

where $\tau = 1$, $0 < \varpi \leq 1$ is the momentum parameter, $\boldsymbol{e}^{(k+1)} \sim N(0, I_{d_{\boldsymbol{z}}})$, and υ_{k+1} is the learning rate. It is worth noting that the algorithm is reduced to SGLD if we set $\varpi = 1$.

In the simulations, we set the learning rate sequence $\{\upsilon_k : k = 1, 2, \ldots\}$ and the step size sequence $\{\gamma_k : k = 1, 2, \ldots\}$ in the forms:

$$\upsilon_k = \frac{C_\upsilon}{c_\upsilon + k^\alpha}, \quad \gamma_k = \frac{C_\gamma}{c_\gamma + k^\alpha},$$

for some constants $C_\upsilon > 0$, $c_\upsilon > 0$, $C_\gamma > 0$ and $c_\gamma > 0$, and $\alpha \in (0, 1]$. The values of C_υ, c_υ, C_γ,c_γ and α used in different experiments are given below.

§3.1 ATE

From the point of view of equation solving, the model (22) in the main text can also be written as

$$y_i = \tau' T_i' + \mu' + \boldsymbol{x}_i \boldsymbol{\beta} + \sigma z_i, \quad i = 1, 2, \ldots, n, \tag{S22}$$

where $T_i' \in \{-1, 1\}$, $\tau' = \tau/2$, and $\mu' = \mu + \tau/2$. Let $\boldsymbol{\theta} = (\tau', \mu', \boldsymbol{\beta}^\top, \sigma)^\top$. Equation (S22) can be solved under the standard framework of EFI. Unless otherwise noted, all results in this paper are based on solving this type of transformed equations.

We set $\alpha = 1/7$ and $\varpi = 0.1$. For $n = 250$, we set $(C_\upsilon, c_\upsilon, C_\gamma, c_\gamma) =$ (200000, 1000000, 54000, 1000000). For $n = 500$ and 1000, we set $(C_\upsilon, c_\upsilon, C_\gamma, c_\gamma) = (500000, 1000000,$ 54000, 1000000).

We set $\mathcal{K} = 5000$ as the number of burn-in iterations, and set $M = 50,000$ as the number of iterations used for fiducial sample collection. We thinned the Markov chain by a factor of $B = 5$; that is, we collected $M/B = 10,000$ samples in each run.

We set $\eta = 500$ and $\epsilon = 1/10$ in construction of the energy function, and perform gradient clipping by norm with 5000 during the first 100 iterations (for finding a reasonably good initial point).

The DNN in the EFI network has two hidden layers, with the widths given by $d_1 = 90$ and $d_2 = 30$, respectively.

§3.2 Linear control response with Non-linear treatment response

We set $\alpha = 1/7$, $\varpi = 0.1$, and $(C_\upsilon, c_\upsilon) = (200000, 1000000)$ and $(C_\gamma, c_\gamma) = (20, 20000)$ for ω_τ^H and $(C_\gamma, c_\gamma) = (20000, 200000)$ for all other parameters. Refer to Figure S1 for the definition of ω_τ^H.

For initialization, we update the $\boldsymbol{w}_n$ with randomly sampled $\boldsymbol{Z}_n^{(t+1)} \sim \pi_0^{\otimes n}(\boldsymbol{Z}_n)$ for the first 5000 iterations. We set $\mathcal{K} = 20,000$ as the number of burn-in iterations, and set $M = 50,000$ as the number of iterations used for fiducial sample collection. We thinned the Markov chain by a factor of $B = 5$; that is, we collected $M/B = 10,000$ samples in each run. We set $\eta = 10$ and $\epsilon = 1/10$ in construction of the energy function, and perform gradient clipping by norm with 5000 during the first 100 iterations.

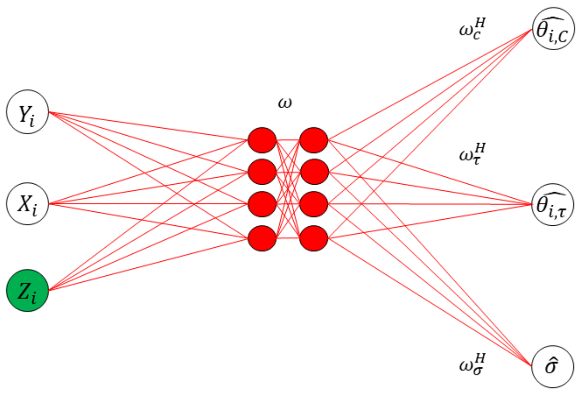

Figure S1: Description of the structure of the DNN model used in the Double-NN method: $\boldsymbol{\theta}$ (i.e., the output layer of the DNN model) can be partitioned into three parts, namely, $\boldsymbol{\theta}_c$, $\boldsymbol{\theta}_\tau$, and σ, where ω_c^L, ω_τ^L and ω_σ^L correspond to the parameters for $\boldsymbol{\theta}_c$, $\boldsymbol{\theta}_\tau$, and $\log(\sigma)$, respectively. In the case that $\boldsymbol{\theta}_c$ or $\boldsymbol{\theta}_\tau$ represents a neural network, we re-scale them by dividing a factor of 25 such that the output values of each output neuron are close to each other, easing the training process.

The DNN in the EFI network has two hidden layers, with the widths given by $d_1 = 90$ and $d_2 = 30$, respectively. The DNN used for modeling the treatment effect has two hidden layers, each hidden layer consisting of 10 hidden neurons.

§3.3 Non-linear control response with Non-linear treatment response

We set $\alpha = 1/7$, $\varpi = 0.1$, and $(C_\upsilon, c_\upsilon) = (500000, 1000000)$ and $(C_\gamma, c_\gamma) = (2.5, 1000000)$ for ω_τ^H and ω_c^H, and set $(C_\gamma, c_\gamma) = (20000, 200000)$ for all other parameters. Refer to Figure S1 for the definitions of ω_τ^H and ω_c^H.

For initialization, we update the $\boldsymbol{w}_n$ with randomly sampled $\boldsymbol{Z}_n^{(t+1)} \sim \pi_0^{\otimes n}(\boldsymbol{z}_n)$ for the first 5000 iterations. We set $\mathcal{K} = 20{,}000$ as the number of burn-in iterations, and set $M = 50{,}000$ as the number of iterations used for fiducial sample collection. We thinned the Markov chain by a factor of $B = 5$; that is, we collected $M/B = 10{,}000$ samples in each run. We set $\eta = 10$ and $\epsilon = 1/10$ in construction of the energy function, and perform gradient clipping by norm with 5000 during the first 100 iterations.

The DNN in the EFI network has two hidden layers, with the widths given by $d_1 = 90$ and $d_2 = 30$, respectively. The DNN used for modeling the treatment effect has two hidden layers, each hidden layer consisting of 10 hidden neurons. The DNN used for modeling the response function under the control has two hidden layers, each hidden layer consisting of 10 hidden neurons.

§3.4 Lalonde

We set $\alpha = 1/7$, $\varpi = 0.1$, and $(C_v, c_v) = (500000, 1000000)$ and $(C_\gamma, c_\gamma) = (2.5, 1000000)$ for ω_τ^H and ω_c^H, and $(C_\gamma, c_\gamma) = (1000, 1000000)$ for all other parameters. Refer to Figure S1 for the definitions of ω_τ^H and ω_c^H.

For initialization, we update the $\boldsymbol{w}_n$ with randomly sampled $\boldsymbol{Z}_n^{(t+1)} \sim \pi_0^{\otimes n}(\boldsymbol{z}_n)$ for the first 10,000 iterations. We set $\mathcal{K} = 20,000$ as the number of burn-in iterations and set $M = 50,000$ as the number of iterations used for fiducial sample collection. We thinned the Markov chain by a factor of $B = 5$; that is, we collected $M/B = 10,000$ samples in each run. We set $\eta = 10$ and $\epsilon = 1/10$ in construction of the energy function, and perform gradient clipping by norm with 5000 for first 100 iterations.

The DNN in the EFI network has two hidden layers, with the widths given by $d_1 = 90$ and $d_2 = 30$, respectively. The DNN used for modeling the treatment effect contains two hidden layers, each hidden layer containing 10 hidden neurons. The DNN used for modeling the response function under the control contains two hidden layers, each hidden layer containing 10 hidden neurons.

§3.5 NLSM

We set $\alpha = 1/7$, $\varpi = 0.1$, and $(C_v, c_v) = (500000, 1000000)$ and $(C_\gamma, c_\gamma) = (2.5, 1000000)$ for ω_τ^H and ω_c^H, and $(C_\gamma, c_\gamma) = (5000, 1000000)$ for all other parameters. Refer to Figure S1 for the definitions of ω_τ^H and ω_c^H.

For initialization, we update the $\boldsymbol{w}_n$ with randomly sampled $Z_n^{(t+1)} \sim \pi_0^{\otimes n}(\boldsymbol{z}_n)$ for the first 10,000 iterations. We set $\mathcal{K} = 20,000$ as the number of burn-in iterations and set $M = 50,000$ as the number of iterations used for fiducial sample collection. We thinned the Markov chain by a factor of $B = 5$; that is, we collected $M/B = 10,000$ samples in each run. We set $\eta = 10$ and $\epsilon = 1/10$ in construction of the energy function, and perform gradient clipping by norm with 5000 for first 100 iterations.

The DNN in the EFI network contains two hidden layers, with the widths given by $d_1 = 90$ and $d_2 = 30$, respectively. The DNN used for modeling the treatment effect contains two hidden layers, each hidden layer containing 10 hidden neurons. The DNN used for modeling the response function under control contains two hidden layers, each hidden layer containing 10 hidden neurons.

References

Dittmer, S., King, E. J., and Maass, P. (2018), "Singular Values for ReLU Layers," *IEEE Transactions on Neural Networks and Learning Systems*, 31, 3594–3605.

Higham, N. J. and Cheng, S. H. (1998), "Modifying the inertia of matrices arising in optimization," *Linear Algebra and its Applications*, 261–279.

Lei, L. and Candès, E. J. (2021), "Conformal Inference of Counterfactuals and Individual Treatment Effects," *Journal of the Royal Statistical Society Series B: Statistical Methodology*, 83, 911–938.

Liang, F., Jia, B., Xue, J., Li, Q., and Luo, Y. (2018a), "An imputation–regularized optimization algorithm for high dimensional missing data problems and beyond," *Journal of the Royal Statistical Society, Series B*, 80, 899–926.

Liang, F., Kim, S., and Sun, Y. (2024), "Exended Fiducial Inference: Toward an Automated Process of Statistical Inference," *Journal of the Royal Statistical Society, Series B*, in press.

Liang, F., Li, Q., and Zhou, L. (2018b), "Bayesian Neural Networks for Selection of Drug Sensitive Genes," *Journal of the American Statistical Association*, 113, 955–972.

Liang, S., Sun, Y., and Liang, F. (2022), "Nonlinear Sufficient Dimension Reduction with a Stochastic Neural Network," *NeurIPS 2022*.

Nielsen, S. (2000), "The stochastic EM algorithm: Estimation and asymptotic results," *Bernoulli*, 6, 457–489.

Sethuraman, M. G., Lopez, R., Mohan, R. V., Fekri, F., Biancalani, T., and Hutter, J.-C. (2023), "NODAGS-Flow: Nonlinear Cyclic Causal Structure Learning," in *International Conference on Artificial Intelligence and Statistics*.

Song, Q. and Liang, F. (2023), "Nearly optimal Bayesian Shrinkage for high dimensional regression," *China Science Mathematics*, 66, 409–442.

Sun, Y. and Liang, F. (2022), "A kernel-expanded stochastic neural network," *Journal of the Royal Statistical Society Series B*, 84, 547–578.

Sun, Y., Song, Q., and Liang, F. (2022), "Consistent Sparse Deep Learning: Theory and Computation," *Journal of the American Statistical Association*, 117, 1981–1995.

Tibshirani, R. J., Foygel Barber, R., Candes, E., and Ramdas, A. (2019), "Conformal Prediction Under Covariate Shift," in *Advances in Neural Information Processing Systems*, eds. Wallach, H., Larochelle, H., Beygelzimer, A., d'Alché-Buc, F., Fox, E., and Garnett, R., Curran Associates, Inc., vol. 32.
